# Gender identity impacts the perception of vocal congruence

**DOI:** 10.3389/fcogn.2026.1638501

**Published:** 2026-03-10

**Authors:** Chiara De Livio, Claudia Mazzuca, Chiara Fini, Anna M. Borghi

**Affiliations:** 1 Department of Psychology, Sapienza University of Rome, Rome, Italy; 2 Department of Dynamic and Clinical Psychology and Health Studies, Sapienza University of Rome, Rome, Italy; 3 Institute of Cognitive Sciences and Technologies, Italian National Research Council, Rome, Italy

**Keywords:** alexithymia, gender identity, inner speech, interoception, TGNC, vocal congruence

## Abstract

This study investigated vocal congruence, i.e., the alignment between self-voice perception and the sense of identity, across cisgender and transgender and gender non-conforming (TGNC) participants (*N* = 44) in three conditions: Silent Reading, Reading Aloud, and Listening to recorded speech. Results revealed that TGNC participants reported significantly lower vocal congruence than cisgender participants across all experimental conditions, with the starkest difference in conditions where auditory feedback was present. This experience of incongruence appears to be modulated by interoceptive sensibility and alexithymia, with TGNC individuals reporting lower interoceptive trust and higher levels of alexithymia. Emotional awareness was positively linked to inner-voice congruence in the TGNC group. Additionally, aspects related to gender-minority stress predicted lower congruence. These findings highlight the complex interplay between gender identity, interoception, emotion regulation strategies, and voice perception.

## 1 Introduction

In the introduction of this paper, we first address how humans' bodily sense of self develops, highlighting the role of interoceptive, social, and environmental aspects. We then argue that the voice constitutes a central sensorimotor trait in which individual and social dimensions intersect, representing a crucial component of self-identity. In this context, we discuss the mechanisms underlying the experience of vocal congruence across different modes of voice perception. Finally, we examine how vocal congruence may vary across populations with different gender identities, which constitutes the primary aim of the present study, and we describe the behavioral self-voice perception paradigm employed to investigate this question.

### 1.1 Bodily self-concept, interoception, and social context

Bodies serve as the primary interface with the external world and constitute the foundation of the human sense of self. Body awareness arises from the ability to integrate multisensory information from the inside and outside of the body (Tsakiris et al., 2011), and the development of body awareness begins with the body ownership, which involves the primal recognition that one's body is the source of sensations, and the sense of agency (Tsakiris et al., 2006). Human sense of body ownership is dynamically influenced by both internal sensations and external interactions (Martel et al., 2016; Tsakiris et al., 2011), and distinguishing between them is a fundamental yet complex task (Zaidel and Salomon, 2023). Interoceptive sensibility can be considered as an expression of a high-level model or “belief” for generating predictions about information coming from inside the body (Barrett and Simmons, 2015; Critchley and Garfinkel, 2017; Pezzulo, 2014; Seth, 2013).

Attention plays a role in interoceptive processes by modulating the flow between top-down prior beliefs and bottom-up signal flow (Seth, 2013). This attentional process on the ongoing bodily experience can be adaptive, but proneness to rumination—i.e., maladaptive repetitive thinking about past events—on somatic cues can lead to maladaptive behaviors (Naraindas et al., 2023; Mehling et al., 2012, 2018). Beyond internal experience, expectations and beliefs also influence body awareness. Beliefs about the body are frequently associated with cultural-specific representations about a desired body (Cattarin et al., 2000; Ricciardelli et al., 2007; Thompson et al., 2005), which might lead to experiences of anxiety and avoidance of social situations.

Novel evidence indeed supports the idea of a deep interconnection between body awareness and self-awareness, and between social interaction and body-awareness (De Vignemont, 2023). Interoception gives the concept of self a solid foundation, enhancing its coherence over time by reducing its susceptibility to external influences (see Arnold et al., 2019 for a review). On the other hand, social situations may reduce interoceptive processing by shifting attention from internally- to externally-focused. Higher interoceptive abilities would mediate the ability to “self-regulate” in social situations by flexibly directing attention toward both internal and external information; the link between interoception and the social self is thus bi-directional (Monti et al., 2022).

### 1.2 The voice as a sensorimotor trait of self and identity

The sound of voice is embroidered in human body schema, and the ability to discriminate between self- and non-self voice cues is fundamental for self-awareness and self-monitoring during verbal communication (Candini et al., 2018; Conde et al., 2018; Fernyhough and Russell, 1997; Graux et al., 2015; Xu et al., 2013). Rather than being just a vehicle of verbal communication, the voice can be considered a salient marker of individuality (Belin et al., 2004; Crow et al., 2021), and centers on a domain where the body intersects with cultural processes of socialization and identity formation (Zimman, 2018). The voice is both an inner bodily signal (i.e., the embodied experience of perceiving oneself speaking) and a means by which individuals transmit information about themselves to interact with others. As such, the voice serves as a bridge between the self and others.

Perceiving one's voice is part of human everyday experience. Specifically, the experience of speech production requires the multimodal integration of different sources of information: auditory, proprioceptive, tactile, and barometric (Kent, 2024; Ito et al., 2009). The internal model of speech, or *somatorepresentation*, reflects how we experience and monitor speech-related sensations (Haggard and de Boer, 2014). This system continually adjusts speech output by comparing expected and actual sensory input, updating its internal models to reduce mismatches (Kent, 2024). This is particularly important given that individuals experience their own voice in multiple, qualitatively distinct ways. These range from everyday overt speech, to the perception of one's voice during playback of recorded speech (e.g., audio messages), to the experience of interacting with themselves through inner speech (Alderson-Day and Fernyhough, 2015; Lœvenbruck et al., 2018; Morin, 2005), or silently reading (Kunz et al., 2025; Perrone-Bertolotti et al., 2012; Vilhauer, 2016; Yao et al., 2011). Despite the differing sensory and cognitive demands of these contexts, individuals typically experience a stable and unified sense of vocal identity.

### 1.3 Vocal congruence and the embodied experience of voice

Although the literature mentioned above has focused on the role of the voice in shaping self-identity and social interactions, not much is known about the mechanism underlying the perception of different instances of one's voice.

A recent survey of 1,522 U.S. participants revealed that nearly 40% of respondents reported discontent with their voice, with no correlation to voice disorders (Naunheim et al., 2023). A recent update (Naunheim et al., 2024) showed an increase in voice complaints from 2012 to 2022, suggesting a possible association between increased voice dissatisfaction and the increased habits of self-recording audio-video contents and remote work, which became widespread globally between and after the COVID-19 pandemic. This incongruence arises from the physical transformation that the voice goes through while speaking: hearing one's voice is mediated both by air and bone conduction, while the voice other people hear is only conducted through air (Orepic et al., 2023; Pörschmann, 2000; Stenfelt, 2016).

In order to define the connection between the embodied experience of having a voice and the auditory experience of hearing one's voice, Crow and colleagues introduced the concept of *vocal congruence*, defined as “…the extent to which one's voice is in alignment, or congruent, with one's sense of self” (Crow et al., 2021, p. 1). To measure this process, they designed a self-report measure of vocal congruence, the Vocal Congruence Scale (VCS). The instrument uses a five-point Likert scale to investigate the degree of individual identification with the voice and the beliefs about the reflection between the voice and the personhood. In addition, the authors showed through a heartbeat detection task (i.e., a classical interoceptive task in which participants are required to judge whether heartbeat sensations are simultaneous with external stimuli presented at different time delays) that the scale has moderate to low correlations with metacognitive judgments of interoceptive awareness and confidence, suggesting that voice perception is related to consciously focusing attention and reflecting upon bodily experience.

This scale captures different faces of the vocal congruence experience, ranging from voice ownership, agency, and control, to functional vocal use and expressiveness in social contexts. Crucially, it addresses identity-related dimensions of vocal congruence such as voice-identity congruence and alignment with one's gender identity. Finally, the scale also investigates metacognitive and evaluative components, including awareness and satisfaction and/or rumination with one's voice. Vocal congruence can thus be understood as a multifaceted phenomenon arising from the perceptual experience of receiving auditory vocal feedback that can match or mismatch with one's intended vocal production. This perceptual mismatch may trigger both an evaluative and metacognitive judgment, as well as an affective response to this experience.

### 1.4 Voice perception and TGNC identities

Gender plays an important role in shaping the individual's bodily experience. Gender identity is typically conceptualized as a person's inherent sense of their own gender, and how they identify as a woman, a man, both, an alternative gender, or neither (American Psychological Association, 2015). Here, in line with previous literature, we use the label TGNC to refer to transgender and gender nonconforming individuals, i.e., people whose gender identity varies from assumptions based on their birth sex (American Psychological Association, 2015). Although this label is not comprehensive, we believe it provides a valuable and respectful shorthand for referring to a diverse range of gender identities.

TGNC individuals face continuous discrimination, inequality, and social stigma in all aspects of their lives (Carmel and Erickson-Schroth, 2016; Connolly et al., 2016; Drabish and Theeke, 2022; Pinna et al., 2022; Romani et al., 2021; Russell and Fish, 2016; Testa et al., 2015; Truszczynski et al., 2022). Previous research highlighted the higher prevalence of alexithymia, body-image disorders, somatization, body uneasiness, and emotion dysregulation within the TGNC population compared to cisgender peers (Budge, 2020; Hatzenbuehler, 2009; Kallitsounaki and Williams, 2023; McGuire et al., 2016; Mirabella et al., 2020, 2024a; Maniaci et al., 2024; Mazzoli et al., 2022; Testa et al., 2015). Body dissatisfaction among individuals with gender incongruence is a major source of distress, encompassing psychological, physical, and biological dimensions and leading to behaviors such as avoidance, body surveillance, and feelings of detachment from the body (Mirabella et al., 2020, 2024a). Discomfort may involve not just secondary sexual characteristics but also non-sexual body parts and may represent a profound concern about gender discrepancies and societal norms (Pu et al., 2025).

The voice is one of the bodily aspects most commonly reported by TGNC people as a source of incongruence and distress (Chadwick et al., 2022; Kennedy and Thibeault, 2020; James et al., 2016; Oestreicher-Kedem et al., 2024; Pu et al., 2025; van de Grift et al., 2016a,b; Ziltzer et al., 2023). Vocal cues can disclose information related to personal identity like provenience, age, or gender, and for TGNC people, they can represent possible unwanted episodes of exposure of their birth sex (Chang and Yung, 2021). TGNC individuals report the need to find and develop a voice and communication that reflect their individual's sense of gender, and often seek speech therapists since their outer voice does not match with their “inner” and “true” voice (Davies et al., 2015). In fact, *gender dysphonia* (i.e., the perception of one's gender identity as being inconsistent with the qualities of one's voice and communication; De Bruin et al., 2000; Kennedy and Thibeault, 2020), has been shown to impact the quality of life and everyday functioning of TGNC individuals (Hancock et al., 2011; Hancock and Pool, 2017; James et al., 2016; dos Santos Oliveira et al., 2024). These aspects intertwine with physiological impairments related to pitch, voice quality, inflections, resonance, and precision in the articulation, intensity, and prosody (Davies et al., 2015), as well as the psychological reactions to them (Hancock and Pool, 2017). Within people identifying as TGNC there is great variation in the extent to which voice changes are undertaken or desired. Some people within this community seek to develop both masculine and feminine speech patterns, either because they identify as bigender or due to external pressures preventing them from fully expressing their gender identity. Others may have a gender identity that does not fit within the traditional woman/man spectrum and desire a more flexible gender presentation.

Culturally inherited stereotypes about what is considered feminine or masculine are often enacted through language (Charlesworth et al., 2021; Jones et al., 2020; Lindqvist et al., 2019; Lewis and Lupyan, 2020; Sczesny et al., 2016; Skewes et al., 2018). Studies with priming paradigms showed that linguistic gender cues can impact both explicit and implicit associations and attitudes of stereotyped group members (Pesciarelli et al., 2019; Steele and Ambady, 2006), and cause self-perception and social behavior to become more congruent to gender stereotypes (Hundhammer and Mussweiler, 2012). However, to date the impact of gendered stereotypes conveyed by language on vocal congruence remains underexplored. Deepening the complex interweaving between the internal bodily experience and cultural constraints might be crucial for the understanding of the TGNC people's experience of gender.

### 1.5 The current study

Little is known about how voice self-perception affects social interactions and, reciprocally, how living in a socio-cultural milieu—where representations of gender identity within a cultural system can shape how individuals perceive themselves—affects the experience of perceiving one's own one voice (Hughes and Harrison, 2013; Peng et al., 2019). To our knowledge, no existing study tackles the relationship between inner and outer voice perception across different gender identities. Furthermore, no research has systematically examined how gender stereotypes regarding the expected sound of female or male voices influence vocal congruence across diverse gender identities. This is striking since the experience of voice incongruence, i.e., the mismatch between the voice perceived while speaking and the actual voice that other people hear (or one's own recorded voice from a voice message), is a common everyday experience also for cisgender people (Holzman and Rousey, 1966).

In this study, we investigate vocal congruence across populations with different gender identities (cisgender and TGNC) using a behavioral self-voice perception task. We build on existing studies of vocal congruence by introducing an additional condition—the perception of inner voice congruence—to previously studied scenarios, such as reading aloud and listening to one's own recorded voice (Crow et al., 2021; Welch and Helou, 2022). Importantly, participants are presented with excerpts of texts conveying either gender-stereotypical content (feminine vs. masculine) or gender-neutral content.

As our main prediction, we hypothesize differences in voice perception between Cisgender and TGNC participants. We expect the TGNC group to experience lower vocal congruence overall, with the lowest scores occurring in conditions that require focus on the externalized voice. Specifically, we expect TGNC participants to experience lower vocal congruence than cisgender participants during the Reading Aloud condition, which involves active speech motor control and simultaneous auditory-somatosensory feedback (including bone conduction). We also expect lower scores for TGNC participants in the Listening condition, which isolates the purely auditory-perceptual evaluation of the self-voice without motor intent. Conversely, we hypothesize that Silent Reading—requiring an engagement of auditory imagery without an external feedback—will elicit relatively higher congruence scores in the TGNC group, as it reflects a mental representation of vocal identity that is less mediated by the physical constraints or acoustic “mismatches” of externalized speech. A summary of the experimental conditions and their underlying processes is provided in Table 1.

**Table 1:** Comparison of experimental conditions by cognitive process and primary feedback.

**Condition**	**Process**	**Primary feedback**
Silent reading	Auditory imagery	None
Reading aloud	Speech production	Auditory (air + bone) & somatosensory
Listening	Auditory perception	Auditory (air only)

Second, to investigate how gendered linguistic primes influence vocal self-perception, we manipulate the semantic content of the texts, presenting excerpts that are gender-stereotypical (masculine or feminine) and gender-neutral. We predict that the semantic content of the texts will interact with participants' gender identity, such that gendered primes will reduce vocal congruence for TGNC individuals by reinforcing stereotypical gender associations. Although a stricter test of the priming effect should have targeted specific gender identities within each group paired with the relevant linguistic stereotype, the scarcity of the sample prevented us from deepening this aspect. Nonetheless, we still expected the gendered semantic content of texts would specifically impact on TGNC participants' vocal congruence perception because it might draw participants' attention to the general gender conceptual dimension.

Prior research suggests that interoceptive sensibility may play an important role in voice perception (Crow et al., 2021; Orepic et al., 2022; Smeltzer et al., 2023). Building on this, and also considering the high prevalence of emotion regulation difficulties—such as alexithymia—among TGNC individuals (Kallitsounaki and Williams, 2023; Mazzoli et al., 2022; Reed et al., 2023), the present study examines a broader set of psychological factors that might influence the experience of vocal congruence across different gender identities. Specifically, we investigate the role of interoceptive sensibility in shaping vocal congruence, as well as the potential disruptive effect of alexithymia and emotion regulation difficulties. In addition we explore the relation between ontological beliefs about gender/sex and vocal incongruence. Finally, we also investigate whether experiences of gender-related discrimination in the TGNC sample influence vocal congruence perception.

### 1.6 Data availability

All the materials, data, scripts, and analyses of the study are available at the OSF repository: https://osf.io/v2gtn/.

## 2 Methods

### 2.1 Participants

A total of 45 participants were recruited for the study. A sensitivity power analysis was conducted using G*Power software (Faul et al., 2007) to determine the minimum detectable effect size given the observed sample size. For the primary Condition × Group interaction, the study had 80% power to detect an effect size of *f* = 0.50 (η^2^ =0.20) at α =0.05. *Post-hoc* approximations for between-group comparisons indicated adequate power to detect medium-to-large effects (*d* = 0.85, power = 87%). After excluding one participant due to failure to complete the questionnaires, the resulting sample size was *N* = 44 for all subsequent statistical analyses (*M Age* = 27.45; *SD Age* = 10.22; Age range = 18–66). We recruited participants through the involvement of an LGBTQIA+ rights local association and a hospital service dedicated to gender-affirmation processes. Trainer vocalists and individuals at advanced stages of the gender-affirmation process who had undergone voice training interventions were not eligible. This was ensured prior to participants' enrollment in the study. We asked for the following demographic information: age, birth sex, gender identity, sexual orientation, educational level, birth country, and languages spoken from childhood. Participants' demographic information is reported in Table 2. Most participants (57%, *n* = 25) were female at birth, whereas 41% (*n* = 18) were male at birth and one participant was intersex. Based on gender identity, we divided participants into two groups: TGNC participants (*n* = 22) and participants who identified as cisgender (*n* = 22). In the cisgender group, 55% of the participants identified as cisgender women (*n* = 12) and 45% as cisgender men (*n* = 10). In the TGNC group, 32% of the participants identified as transgender men (*n* = 7), 23% as transgender women (*n* = 5), and 23% as non-binary (*n* = 5). Four participants (18%) used the “other” response option and reported they identified as “*genderfluid transgender man*,” “*non-binary transgender man*,” “*genderfluid*,” “*woman*.” The two groups were comparable in terms of age, *t*_(36.33)_ = −0.087, *p* = 0.930 (*M* cisgender = 27.59; *SD* = 8.04; *M* TGNC = 27.31; *SD* = 12.21).

**Table 2:** Socio-demographic information of the sample (*n* = 44).

**Birth sex**	***n* (%)**	**Gender identity**	***n* (%)**	**Education**	***n* (%)**	**Birth Country**	***n* (%)**	**Italian proficiency**	***n* (%)**
Female	25 (57%)	Cisgender woman	13 (30%)	Second primary school	4 (9%)	Italy	43 (99%)	First language	44 (100%)
Male	18 (41%)	Cisgender man	10 (23%)	High school	19 (43%)	Romania	1 (1%)	Other languages spoken from childhood	3 (7%)
Intersex	1 (2%)	Non-binary	5 (11%)	Bachelor degree	4 (9%)				
		Transgender woman	5 (11%)	Master degree	11 (25%)				
		Transgender man	7 (16%)	PhD	6 (14%)				
		Other	4(9%)						
									

In the cisgender group, one participant completed only middle school (5%), 36% (*n* = 8) of participants reported having a high school diploma, one participant obtained a bachelor degree (5%), 36% (*n* = 8) a master's degree, and 18% (*n* = 4) a PhD title. In the TGNC group, 14% (*n* = 3) completed middle school, 50% (*n* = 11) of participants reported having a high school diploma, 36% (*n* = 3) had a bachelor's degree, 14% (*n* = 3) had a master's degree, and 18% (*n* = 4) a PhD title (Table 2).

### 2.2 General procedure

The experimental procedure was divided into three parts, and participants completed it in two sessions. In the first session, participants completed the voice perception task to assess levels of vocal congruence. As an additional, separate part of the experimental procedure, participants also rated a set of words onn multiple semantic dimensions. However, these results are not discussed here, as they are not central to the aims of this study. Finally, they gave demographic information. In the second session, participants completed questionnaires investigating interoceptive sensibility, emotion regulation, alexithymia, gender identity, and gender discrimination. Questionnaires were administered 2 days after the completion of the first session to avoid fatigue and carryover effects of the voice perception task. The experimental procedure was implemented with Qualtrics, using an on-line questionnaire divided into sections that participants filled in a fixed order. The study obtained ethical approval from the Ethics Committee of the Sapienza University of Rome (Ethical Approval 0000856).

### 2.3 Session 1: vocal congruence assessment

#### 2.3.1 Materials

Four texts were chosen based on the presence or absence of a clear gender stereotypic representation of feminine and masculine gender identities. We selected the two gender-neutral texts from “Come vivevano i Greci” (Paoli, 1957) and from “Un mare di silenzio” Rava, (2012), and the two gendered texts from “L'abbecedario degli Stereotipi di Genere” (Priulla and Sammartino, 2020). Importantly, the former excerpts were selected from educational materials aimed at lower secondary school students, characterized by accessible language and designed to assess reading comprehension, while the latter were taken from an Italian educational booklet specifically dealing with gender stereotypes. In the gendered texts, stereotypical descriptions of gender identity and socialization for both feminine and masculine gender identities were presented to the participants. For example, the female text stated: “*Even before children are born, many parents tend to adopt different attitudes based on the child's sex. If they believe it will be a girl, she is often imagined as gentle, sensitive, and naturally inclined toward relationships and married life*.” The male text stated: “*Even before children are born, many parents tend to adopt different attitudes based on the expected sex of the child. If they believe it will be a boy, they often imagine him as athletic, success-driven, strong, and independent*.” All the texts had the same length (see Supplementary materials).

- *Vocal Congruence Scale* (Crow et al., 2021). We administered a self-report questionnaire (0 = strongly disagree; 4 = strongly agree) designed to measure vocal congruence. The questionnaire was back-translated, and the scoring was adapted for this study (1 = strongly disagree; 5 = strongly agree). The scale comprises 10 items assessing various facets of the alignment between the voice and the self, including voice ownership, functional control, voice-identity congruence, voice agency and control, satisfaction, awareness/rumination about one's voice, voice clarity, communicative effectiveness, emotional expression, and gender congruence (see Table 3). However, the original validation study did not report distinct subscales or a factor structure supporting the delineation of separate subdimensions. Therefore, we treated it as a unidimensional measure and calculated the total sum score of all items.
- *Inner-Outer Voice Congruence Rating*. To measure the effect of the type of text manipulation, after each text we administered a five-points Likert scale rating to test the congruency between the inner voice perceived during the Silent Reading and the voice heard during the Listening condition. For instructions about the rating scale, see Table 4.

**Table 3:** Vocal congruence scale.

**Italian translation**	**Original English version (Crow et al., 2021)**
*Nel compito di produzione che ho appena terminato…* …*sembrava che la mia voce appartenesse a me*.	In the speaking/listening task I just performed… …it seemed like my voice belonged to me.
…*sembrava che la mia voce funzionasse normalmente*.	… it seemed like my voice was functioning as it normally does.
…*sembrava che la mia voce riflettesse chi sono*.	… it seemed like my voice reflected who I am.
…*sembrava avessi il controllo della mia voce*.	… it seemed like I was in control of my voice.
…sono soddisfatt^*^ di come suonava la mia voce.	… I am satisfied with how my voice sounded.
…*stavo pensando al modo in cui suonava la mia voce*.	… I was thinking about the way my voice sounded.
…*mi sento come se la mia voce suonasse chiara*.	… I feel as though my voice sounded clear.
…*mi sento come se stessi parlando in modo facilmente comprensibile*.	… I feel as though I was speaking in a way that I was easily understood.
…*mi sento come se la mia voce riflettesse accuratamente il mio stato d'animo*.	… I feel as though my voice accurately reflected my mood.
…*mi sento come se la mia voce riflettesse il mio genere*.	… I feel as though my voice reflected my gender.
*1, Fortemente in disaccordo; 2, In disaccordo; 3, Neutrale; 4, In accordo; 5, Fortemente in accordo*.	0, Strongly disagree; 1, Disagree; 2, Neutral; 3, Agree; 4, Strongly agree.

The left column presents the Italian version of the scale, back-translated and adapted by the authors taking care of ensuring gender-inclusive language through the use of the desinence. ^*^see e.g., Mirabella et al. (2024b); the right column shows the original English version (Crow et al., 2021).

**Table 4:** Instructions for the inner-outer voice congruence rating.

**Original Italian version**	**English translation**
*Quanto ti sembra che la tua voce come la percepisci internamente corrisponda alla voce registrata? (1 = Per Nulla; 5 = Completamente)*	How much does your voice as you perceive it internally match your recorded voice? (1 = not at all; 5 = completely)

#### 2.3.2 Procedure

Participants were seated in front of a computer for the duration of the session. All recordings were carried out in person in a quiet room. Voice data were recorded using the built-in microphone of an HP Specter ×360 laptop, which was used for all participants to maintain consistency across recordings. Before each session, an audio check was conducted to ensure clear signal quality without distortion or significant background noise. All recordings were played back on the same laptop using standardized volume settings for every participant.

Participants were presented with four texts (Gender-neutral text 1, Stereotypically Masculine text, Gender-neutral text 2, Stereotypically Feminine text) in a fixed order to avoid any carryover effects of gender manipulation. They had to complete a Silent Reading, a Reading Aloud and a Listening task for each text. The order of the three conditions was kept fixed to avoid carryover effects of both Reading Aloud and Listening conditions on the Silent Reading condition. In the Silent Reading condition, participants were instructed to read the text silently without time pressure. In the Reading Aloud condition, they were asked to read the exact text aloud and to record themselves while reading by using the online questionnaire's interface. In the Listening condition, participants were asked to listen to their recorded voice; they could stop listening at any moment. After each condition, participants were presented with an Italian version of the Vocal Congruence Scale (Crow et al., 2021; see Table 3). At the end of each text block, they were asked to rate the perceived congruence between the inner voice heard during the Silent Reading and the voice heard during the Listening condition (see Table 4). We added this explicit question to understand better the priming role of gender-stereotyped text in the experience of vocal congruence. After the experimental task, participants provided their socio-demographic information. Finally, the questionnaires were administered 2 days after.

### 2.4 Session 2: questionnaires

#### 2.4.1 Materials and procedure

In the last session, held 2 days after the vocal congruence assessment, all the participants were asked to complete the following set of questionnaires via Qualtrics: (a) *The Multidimensional Assessment of Interoceptive Awareness* (Mehling et al., 2012) to investigate the potential influence of interoceptive sensibility on vocal congruence; (b) the Emotion Regulation Questionnaire (Gross and John, 2003), to assess participants' typical cognitive strategies for managing emotions; (c) the Toronto Alexithymia Scale (Bagby et al., 1994), to control for individual differences in alexithymia; and (d) the Multi-Gender Identity Questionnaire (Joel et al., 2014) to explore the degree of binary vs. flexible gender identity among participants. Additionally, TGNC participants completed the Italian adaptation of the Italian adaptation of the Gender Minority Stress and Resilience Measure (Scandurra et al., 2020; Testa et al., 2015; Italian) assessing gender-related discrimination, as well as the Italian versions of the Italian adaptation of the Transsexual Voice Questionnaire for Male-to-Female Transsexuals (TVQ^MtF^, Dacakis et al., 2013) and Transsexual Voice Questionnaire for Female-to-Male Transsexuals (TVQ^FtM^, Dacakis et al., 2013; Kreukels et al., 2012) to further investigate voice-related functioning in transgender women and men, respectively.

While a broader set of questionnaires was administered, the analyses presented in this work focuses on four measures described below—MAIA, TAS-20, MULTI-GIQ, and GMSR—selected a priori for their relevance to the research questions.

- *The Multidimensional Assessment of Interoceptive Awareness* (Mehling et al., 2012). We used this 32-items self-report questionnaire, which assesses interoceptive awareness across eight constructs: (i) *Noticing*: awareness of uncomfortable, comfortable, or neutral body sensations; (ii) *Non-Distracting*: avoiding distraction to cope with bodily discomfort; (iii) *Not-Worrying*: tendency not to experience emotional distress about bodily discomfort; (iv) *Attention Regulation*: ability to sustain and control attention on the body; (v) *Emotional Awareness*: internal process involving the ability to attribute specific physical sensations to physiological manifestations of emotions; (vi) *Self-Regulation*: ability to regulate distress by attention to body sensations; (vii) *Body-Listening*: tendency to actively attend to body signals for insights; (viii) *Trust*: experience of one's body as safe and trustworthy. Each construct is rated on a scale from 0 to 5, with higher scores indicating greater interoceptive awareness. Not-Distracting and Not-Worrying sub-scales have reverse scored items. For the purpose of this study, we used the Italian adaptation of the MAIA questionnaire (Calì et al., 2015).
- *The Toronto Alexithymia Scale* (Bagby et al., 1994). We assessed alexithymia with the Italian version (Bressi et al., 1996) of the 20-item Toronto Alexithymia Scale (TAS-20; Bagby et al., 1994). The TAS-20 is a 5 points Likert scale comprising three subscales: (i) difficulty identifying feelings (DIF) measures an individual's ability to recognize and identify their own emotional states; (ii) difficulty describing feelings (DDF) assesses the ability to verbally express and describe their emotions; (iii) externally oriented thinking (EOT) gauges the tendency to focus attention externally rather than internally. While factor subscales have been used in previous studies, recent research favors a single total score (Bagby et al., 2020).
- *The Multi-Gender Identity Questionnaire* (Joel et al., 2014). The Multi-Gender Identity Questionnaire (Multi-GIQ) consists of 24 questions, some of which are gender-neutral and others that are specifically designed for women and men participants. Answers were marked over a five-point Likert scale ranging from “Never” (0) to “Always” (4). A “Not relevant” item is present where necessary (e.g., the question: “In the past 12 months, have you had the wish or desire to be a man?” is not relevant for men). The scale investigates the following nine dimensions: (i) Feeling as a woman (Q3, Q14); (ii) Feeling as a man (Q4, Q13); (iii) Feeling as both genders (Q15, Q16); (iv) Feeling as neither gender (Q17); (v) Satisfaction being the affirmed gender (Q2 and Q1 for men and women, respectively); (vi) Wishing to be the “other” gender (Q21 and Q20 for men and women, respectively); (vii) Dislike of the sexed body (Q23 and Q22 for male- and female-assigned participants, respectively); (viii) Wishing to have the body of the “other” sex (Q24). Questions Q11 and Q12, pertain to buying and wearing clothes of the “other” sex. For the purpose of this study, the questionnaire was back-translated in Italian.

In addition to these, we also presented the TGNC group with the Italian adaptation of the *Gender Minority Stress and Resilience Measure* (Testa et al., 2015; Italian: Scandurra et al., 2020). The GMSR scale is composed of 58 Likert-scaled items' and measures distal stressors (discrimination, rejection, victimization, and non-affirmation), proximal stressors (internalized transphobia, negative expectations, and non-disclosure), and resilience factors (pride and community connectedness) TGNC. The scale is composed of a nine-factors: Gender-Related Discrimination (five items), Gender-Related Rejection (six items), Gender-Related Victimization (six items), Non-affirmation of Gender Identity (six items), Internalized Transphobia (eight items), Pride (eight items), Negative Expectations for Future Events (nine items), Non-disclosure (five items), and Community Connectedness (five items).

## 3 Data analysis

Data preprocessing, analysis, and visualization were conducted using R (R Core Team, 2023) within the RStudio environment (Posit Team, 2025). Data preprocessing was performed using the “dplyr” package (Wickham et al., 2023), and data distributions were visualized using the “ggplot2” package (Wickham, 2016).

### 3.1 Vocal congruence task

The distribution of VCS total scores was assessed using visual inspection of histograms and Q–Q plots alongside formal normality tests. The distribution appeared approximately unimodal and bell-shaped, with observations primarily concentrated in the mid-range. While the Shapiro-Wilk test (via “*stats*” R's Package) indicated a statistically significant deviation from normality (*W* = 0.9746, *p* < 0.001), visual diagnostics revealed that this deviation was primarily confined to the extreme tails (please refer to Supplementary materials), reflecting the bounded nature of the 10–50 sum score.

Given that linear mixed-effects models are robust to moderate departures from normality (Schielzeth et al., 2020), VCS total scores were analyzed using linear mixed-effects models (via the “*lme4*” package in R; Bates et al., 2015).

As a preliminary step, we conducted within-participant comparisons to assess whether the two gender-neutral texts differed in their effects on vocal congruence, and whether the two gendered texts differed from each other. Given the non-normal distribution of vocal congruence sum scores, we used Wilcoxon rank-sum tests with continuity correction. Results indicated no significant difference between the two gender-neutral texts (*W* = 8,529.5, *p* = 0.769), nor between the two gendered texts (*W* = 8,671.5, *p* = 0.948).

Because the primary level of analysis in the present study contrasts neutral vs. masculine vs. feminine texts, and only the neutral condition included two empirically equivalent texts, we averaged vocal congruence scores across the two neutral texts within participants. This approach preserves all available data while ensuring that the neutral condition contributes to a single, stable estimate per participant, comparable to the masculine and feminine conditions.

#### 3.1.1 Model structure

Vocal Congruence Scale sum scores for each Type of Text and for each Condition were used for the main model as the dependent variable. Group (TGNC vs. cisgender), Type of Text (Averaged Gender-neutral vs. Stereotypically Feminine vs. Stereotypically Masculine), Condition (Silent Reading vs. Reading Aloud vs. Listening), and their three way interaction were inserted in the model as fixed factors. To account for inter-individual variability in baseline vocal congruence, we included participants as a random intercept (1| Participant). *Post-hoc* contrasts were carried out with the “*emmeans*” R's package (Lenth, 2024) using Tukey's adjustment for multiple comparisons.

Model selection was performed by comparing an additive model (Group + Condition + Text) against a full factorial model including interactions (Group ^*^ Condition ^*^ Text). A Likelihood Ratio Test indicated that the inclusion of interaction terms significantly improved model fit, $\chi_{\left(12\right)}^{2}$ = 51.38, *p* < 0.001, *AIC no interaction* = 2,531.6, *AIC interaction* = 2,504.2, suggesting that the added complexity was justified by the increased explanatory power.

Model assumptions were verified using the “*DHARMa*” R package (Hartig, 2024) to assess scaled residuals, which revealed no significant evidence of overdispersion, outliers, or deviations from the expected distribution (please refer to Supplementary materials).

To control for potential order effects resulting from the fixed sequence of conditions and texts, we included global trial order as a covariate in a supplementary linear mixed-effects model, in interaction with the Group. The inclusion of trial order did not significantly improve model fit, $\chi_{\left(2\right)}^{2}$ = 1.856, *p* = 0.395. Results revealed a non-significant negative main effect of trial order (β = −0.13, *SE* = 0.09, *p* = 0.175). Also the interaction between trial order and Group was non-significant (β = 0.14, *SE* = 0.13, *p* = 0.309). This suggests that the fixed order did not differentially impact the groups or bias the primary comparisons of interest.

### 3.2 Inner-outer voice congruence rating

Prior to any statistical analysis, we examined distributional assumptions. Shapiro-Wilk tests revealed that the distribution significantly deviated from normality (*W* = 0.884, *p* < 0.001). Visual inspection of histograms confirmed a non-normal distribution with bimodal tendencies (peaks at ratings 1–2 and 3–4).

To further investigate vocal congruence as a function of Type of Text, we initially fitted a cumulative link mixed model with the “*ordinal*” R's package (Christensen, 2022) featuring scores of the Inner-Outer Voice Congruence Rating as dependent variable, Group (TGNC vs. cisgender), Type of Text (Gender-neutral vs. Stereotypically Feminine vs. Stereotypically Masculine), and their interaction as fixed factors, and participants as random intercepts. However, the model failed to converge due to quasi-separation issues in the data.

Therefore, we relied on non-parametric testing. To compare ratings between TGNC and cisgender participants across text types, we conducted Wilcoxon rank-sum tests with Bonferroni correction via the “*rstatix*” package (Kassambara, 2023).

### 3.3 Questionnaires

To account for the potential difference in the questionnaires (MAIA, TAS-20, MULTI-GIQ, GMSR) scores between the two groups, we employed one-way ANOVAs. We further examined the role of each variable targeted by questionnaires (interoceptive sensibility, alexithymia, gender identity, gender-related discrimination) on vocal congruence. Given the exploratory nature of these analyses across multiple psychological domains, we ran separate linear mixed models for each subscale, using the same structure and methodology as the main model. To address the risk of Type I errors associated with multiple testing, we applied the Benjamini-Hochberg False Discovery Rate (FDR) correction to all *p*-values across the total set of models. *Post-hoc* contrasts were conducted with the “*emtrends*” function from the “*emmeans*” R package.

We investigated whether individual differences in the general ability to attend to and connect with bodily sensations—measured by the MAIA scale—and the ability to identify and express feelings—assessed via the TAS-20 scale—may moderate the effect of the experimental manipulation. Furthermore, we explored how aspects related to gender identity representation, as captured by the MULTI-GIQ, might affect judgments of vocal congruence. Finally, we examined whether experiences of gender discrimination, measured by the GMSR questionnaire, might specifically impair vocal congruence in TGNC participants. In the analyses we will only focus on the impact of each covariate on vocal congruence and its interaction with the different groups as a function of experimental conditions. For the full set of additional models please refer to the Supplementary materials.

Like for the previous vocal congruence model, we compared for each model featuring covariates the full model with the three-way interaction to a reduced model with only the two-way interaction between Condition and Group. Since the full model did not significantly improve the fit, $\chi_{\left(12\right)}^{2}$ = 7.92, *p* = 0.791, and had higher AIC and BIC values (AIC = 2.504 vs. 2,488; BIC = 2,583 vs. 2,519), we retained the two-way interaction model for each covariate.

## 4 Results

### 4.1 Vocal congruence task

**Figure 1:**
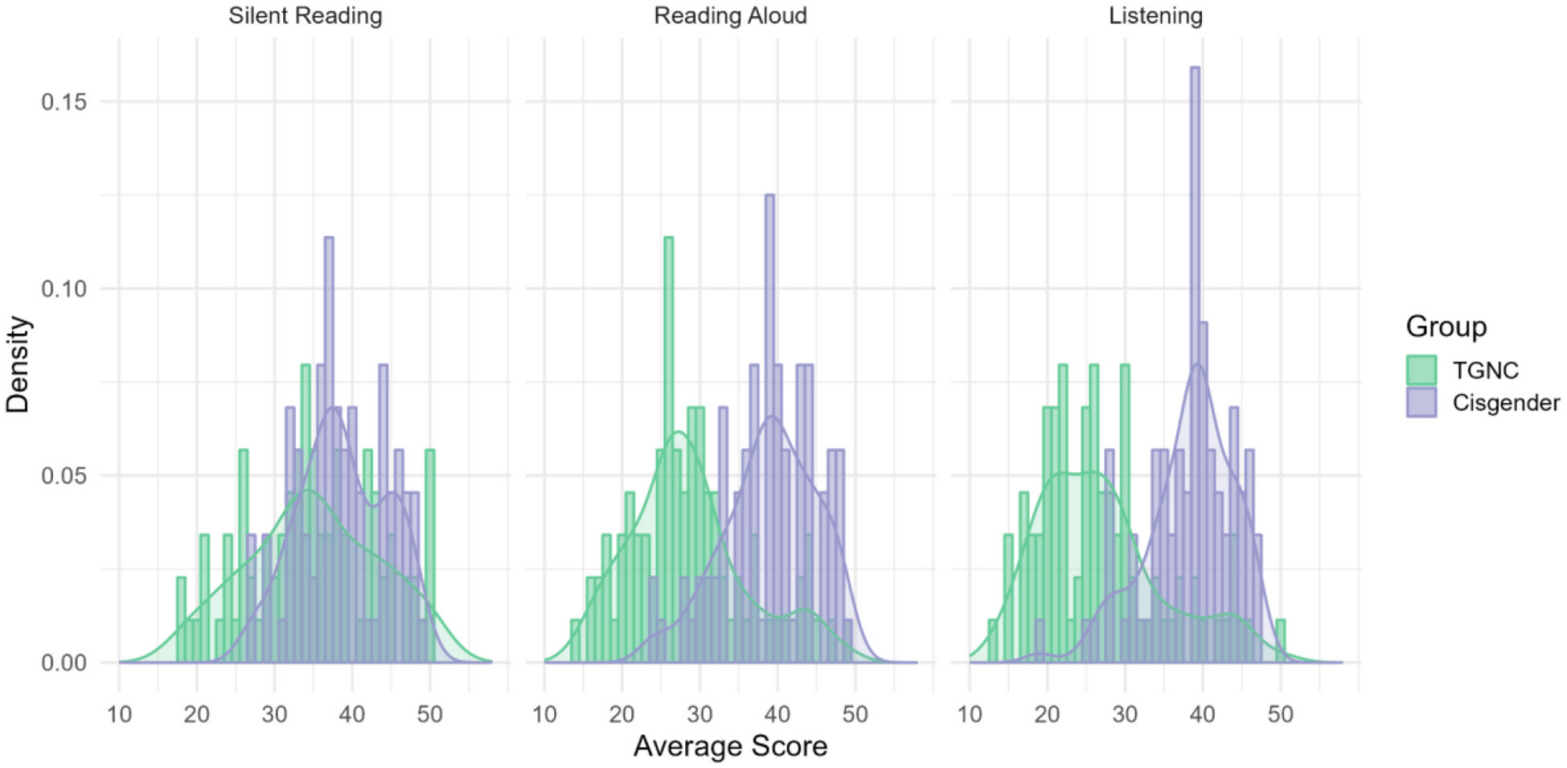
Density distributions of VCS average scores across conditions and groups. This figure presents density distributions of average scores across the three conditions (silent reading, reading aloud, and listening) and groups (TGNC vs. Cisgender).

Density distributions of average VCS scores across Conditions and Groups suggest distinct performance profiles. Cisgender participants consistently exhibit slightly higher peak densities and appear to have somewhat more concentrated distributions around higher average scores. The TGNC group demonstrates more varied distribution patterns across modalities, with the Listening condition showing the most distinct separation between the groups (see Figure 1).

Our first research question investigates whether linguistic gender stereotypes affect the perception of vocal congruence in different conditions as a function of different gender identity experiences.

Looking at intra-class correlations, approximately 51.5% of the total variance in VCS Total scores was attributable to stable differences between participants.

**Figure 2:**
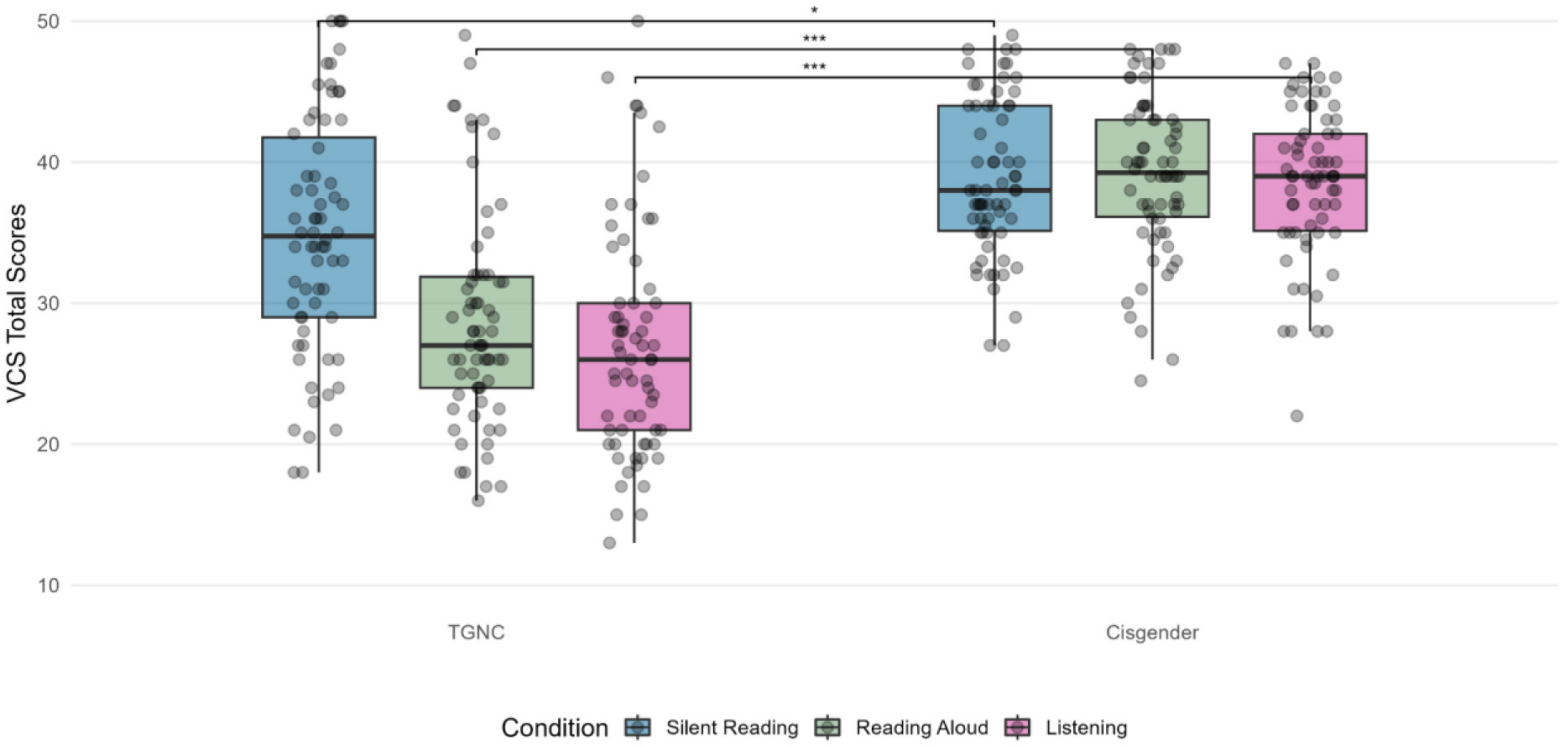
Interaction plots of VCS total scores by condition and group. Distribution of predicted vocal congruence scale (VCS) total scores across the three conditions (silent reading: blue; reading aloud: green; listening: pink) for TGNC and Cisgender participants. Thick black dots represent estimated marginal means and their standard errors (vertical thick black line), and dots represent raw data. ^*^ indicates *p* < .05; ^**^ indicates *p* < .01; ^***^ indicates *p* < .001.

The three-way interaction among Condition, Group, and Type of Text was not significant, $\chi_{\left(4\right)}^{2}$ = 0.34, *p* = 0.986. There was, however, a significant two-way interaction between Condition and Group, $\chi_{\left(2\right)}^{2}$ = 47.24, *p* < 0.001. Pairwise comparisons show that the two groups differed in the Silent Reading condition, β = −3.91, *t* = −2.250, *SE* = 1.74, *p* = 0.028, with TGNC participants reporting lower vocal congruence compared to cisgender participants (*EMM TGNC* = 34.9, *SE* = 1.22, UCL = 32.5, LCL = 37.4; *EMM Cisgender* = 38.8, *SE* = 1.23, LCL = 36.4, UCL = 41.3). Likewise, the two groups scores also differed in the Reading Aloud condition, β = –10.55, *t* = −6.070, *SE* = 1.74, *p* < 0.001, with TGNC participants reporting significantly lower vocal congruence compared to cisgender participants (*EMM TGNC* = 28.7, *SE* = 1.23, LCL = 26.2, UCL = 31.1; *EMM Cisgender* = 39.2, *SE* = 1.23, LCL = 36.8, UCL = 41.7). Finally, the two groups differed in the Listening condition too, β = –11.48, *t* = −6.606, *SE* = 1.74, *p* < 0.001, with TGNC participants reporting significantly lower vocal congruence compared to cisgender participants (*EMM TGNC* = 27.1, *SE* = 1.23, LCL = 24.6, UCL = 29.5; *EMM Cisgender* = 38.6, *SE* = 1.23, LCL = 36.1, UCL = 41.0). Finally, TGNC participants gave higher scores of vocal congruence in the Silent Reading compared to the Reading Aloud, β = 6.22, *t* = 7.334, *SE* = 0.84, *p* < 0.001, and Listening conditions, β = 1.61, *t* = 9.234, *SE* = 0.84, *p* < 0.001, while there was no difference between the Reading Aloud condition and the Listening condition, β = 1.61, *SE* = 0.84, *p* = 0.141 (*EMM Silent Reading* = 33.9, *SE* = 1.23, LCL = 32.5, UCL = 34.7; *EMM Reading Aloud* = 28.7, *SE* = 1.23, LCL = 26.2, UCL = 31.1; *EMM Listening* = 27.1, *SE* = 1.23, LCL = 24.6, UCL = 29.5). No significant difference emerged across conditions for the cisgender group, all *p*_s_ ≥ 0.701 (see Figure 2).

The model additionally indicated significant main effects of Condition, $\chi_{\left(2\right)}^{2}$ = 48.52, *p* < 0.001, and Group, $\chi_{\left(2\right)}^{2}$ = 29.44, *p* < 0.001, while Text was not significant, $\chi_{\left(2\right)}^{2}$ = 2.08, *p* = 0.352. No other main effects or interactions reached significance (all *ps* > 0.218; please refer to Supplementary materials for report of model results).

### 4.2 Inner-outer congruence rating

**Figure 3:**
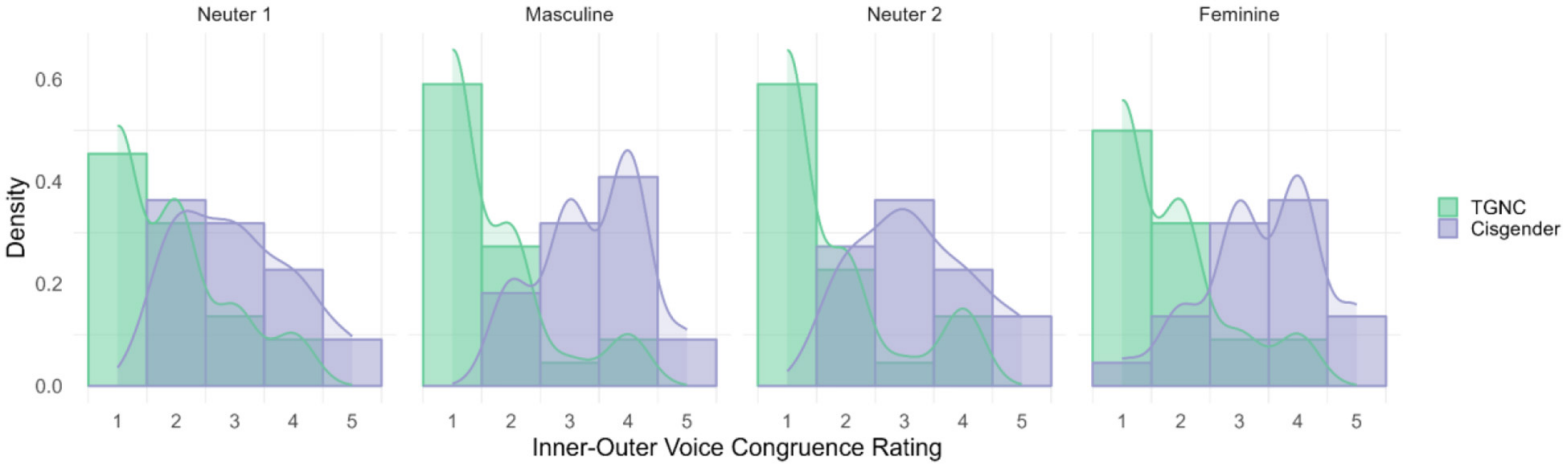
Density distributions for the inner-outer voice congruence rating across type of texts and groups. Density distributions of participants' responses to “How closely does your voice as you perceive it internally correspond to the recorded voice,” averaged for texts types (gender-neutral, stereotypically feminine, stereotypically masculine) and groups (TGNC vs. Cisgender). The scale was administered on five-point Likert scales ranging from 1 = “*not at all*” to 5 = “*completely*”.

Density distributions reveal that TGNC participants display greater variability compared to Cisgender participants, and reported consistently lower alignment between internal and external voice perception (Figure 3).

Wilcoxon rank-sum tests revealed significant differences between TGNC and cisgender participants across all text, all *ps* ≤ 0.002 (Bonferroni adjusted, see Table 5).

**Table 5:** Descriptive statistics (means and standard deviations) of inner-outer voice congruence rating scores for Cisgender and TGNC participants along with Wilcoxon Rank-Sum Tests results comparing their judgments.

**Text type**	***M* Cisgender**	***SD* Cisgender**	***M* TGNC**	***SD* TGNC**	***W***	***P* adj**
Neuter 1	3.04	0.99	1.86	0.99	97.50	0.002
Masculine	3.40	0.90	1.63	0.95	50.50	<0.001
Neuter 2	3.22	1.02	1.72	1.07	74.50	<0.001
Feminine	3.40	1.05	1.77	0.97	68.00	<0.001

Descriptive statistics averaging the two groups ratings across conditions further evidenced the lower perceived congruence between TGNC's group inner vs. outer voice (*M* TGNC = 1.75; *SD* = 0.98; *M* Cisgender = 3.27; *SD* = 0.99).

Overall, the results tackling vocal congruence as emerging from the analyses of both the Vocal Congruence Scale and the Inner-Outer Congruence Rating suggest that TGNC participants experience vocal congruence in an idiosyncratic and subjective manner. Specifically, in the VCS assessing more general aspects connected to voice representation (e.g., control, adherence to identity, satisfaction, and awareness) the two groups differed in all conditions (i.e., Silent Reading, Reading Aloud, and Listening)—showing a decreasing perceived congruence for the TGNC group as a function of whether the condition required an auditory feedback or not. Hence, we found the starkest difference between the two groups in the Listening condition. The internal representation of their speech from the early stage (Silent Reading) to the latest stages of articulatory and/or auditory components (respectively, the Reading Aloud and Listening condition) would be perceived as not reflecting features of the bodily self. Moreover, the rating task investigating the perceived mismatch between one's own inner vs. outer voice highlighted the two groups systematically diverged in their assessments—with the TGNC group once again reporting lower congruence compared to the cisgender group, regardless of the experimental condition.

### 4.3 Questionnaires: relationship with vocal congruence

The previous analyses showed that vocal congruence varies across gender identities, with transgender and gender non-conforming (TGNC) participants systematically reporting lower congruence compared to cisgender participants. To better understand this phenomenon, we fitted separate models investigating the impact of specific aspects related to interoceptive and metacognitive-emotional components as well as aspects related to gender identity on vocal congruence (see Data Analysis). For space reasons, below we only report results from models in which the interaction between specific covariates, experimental condition, and group—where relevant—is significant. For the complete report of results please refer to Supplementary materials.

#### 4.3.1 Interoception

##### 4.3.1.1 Interoceptive sensibility

The two groups differed on two out of the 10 subscales of the MAIA questionnaire (see Supplementary materials for all the results). Specifically, TGNC participants gave lower scores of Self-Regulation compared to cisgender participants, *F*_(1)_ = 10.1, *p* = 0.002, *M* TGNC = 2.29; *SD* = 1.25; *M* cisgender = 3.34; *SD* = 0.90. In addition, TGNC participants also gave lower scores of Trust compared to cisgender participants, *F*_(1)_ = 35.8, *p* < 0.001, *M* TGNC = 2.68; *SD* = 1.22; *M* Cisgender = 4.56; *SD* = 0.83.

###### 4.3.1.1.1 Emotional awareness

We observed a significant three-way interaction between Group, Condition, and Emotional Awareness, $\chi_{\left(2\right)}^{2}$ = 21.34, *p* adj < 0.001. Estimated trends showed that for the TGNC group higher VCS scores in the Silent Reading condition are associated with significantly higher scores of Emotional Awareness (β *TGNC* = 2.84, *SE* = 1.06, *t* = 2.687, LCL = 0.71, UCL = 4.97, *p* = 0.009). No other significant comparisons reached significance, all *ps* ≥ 0.591.

The results also indicated a significant two-way interaction between Condition and Emotional Awareness, $\chi_{\left(2\right)}^{2}$ = 18.81, *p* adj < 0.001, as well as a significant two-way interaction between Condition and Group, $\chi_{\left(2\right)}^{2}$ = 62.62, *p* adj < 0.001. Finally, the model yielded to significant main effects for Condition, $\chi_{\left(2\right)}^{2}$ = 69.88, *p* adj < 0.001, and Group, $\chi_{\left(1\right)}^{2}$ = 27.81, *p* adj < 0.001. No main effect of Emotional Awareness was detected, $\chi_{\left(1\right)}^{2}$ = 0.52, *p* adj = 0.546, all other *ps* adj > 0.759.

###### 4.3.1.1.2 Not worrying

The model yielded significant three-way interaction between Condition, Group, and Not-Worrying, $\chi_{\left(1\right)}^{2}$ = 8.41, *p* adj = 0.026. Estimated linear trends showed that for TGNC Group higher scores on the VCS scores in the Reading Aloud Condition are associated with significantly higher scores of Not Worrying (β *TGNC* = 3.05, *SE* = 0.97, *t* = 3.142, LCL = 1.10, UCL = 5.00, *p* = 0.002). In addition, for TGNC Group higher scores on the VCS in the Listening Condition are associated with significantly higher scores of Not Worrying (β *TGNC* = 3.58, *SE* = 0.92, *t* = 3.684, LCL = 1.63, UCL = 5.53, *p* < 0.001). No other significant comparison emerged, all *ps* ≥ 0.191.

The results also indicated a significant two-way interaction between Condition and Group, $\chi_{\left(1\right)}^{2}$ = 57.44, *p* adj < 0.001, as well as significant main effects of Group, $\chi_{\left(1\right)}^{2}$ = 29.56, *p* adj < 0.001, and Condition, $\chi_{\left(2\right)}^{2}$ = 61.64, *p* adj < 0.001. No other two-way interactions or main effects reached significance, all *ps* adj ≥ 0.060.

###### 4.3.1.1.3 Trusting

We observed a significant three-way interaction between Group, Condition, and Trusting, $\chi_{\left(2\right)}^{2}$ = 19.91, *p* adj < 0.001. Estimated linear trends showed that for the TGNC Group higher VCS scores in the Reading Aloud Condition are associated with significantly high scores of Trust (β *TGNC* = 3.14, *SE* = 0.99, *t* = 3.181, LCL = 1.16, UCL = 5.13, *p* = 0.002). Moreover, for the TGNC Group higher VCS scores in the Listening Condition are associated with significantly high scores of Trust (β *TGNC* = 3.19, *SE* = 0.99, *t* = 3.222, LCL = 1.20, UCL = 5.17, *p* = 0.002). No other comparison reached significance, all *ps* > 0.216.

The results also indicated a significant two-way interaction between Condition and Trusting, $\chi_{\left(2\right)}^{2}$ = 29.67, *p* adj < 0.001. For this model, the two-way interaction between Condition and Group was not significant, $\chi_{\left(2\right)}^{2}$ = 5.06, *p* adj = 0.124. We observed significant main effects for Condition, $\chi_{\left(2\right)}^{2}$ = 11.18, *p* adj = 0.007, and Group, $\chi_{\left(1\right)}^{2}$ = 9.74, *p* adj = 0.003. The main effect of Trusting was not statistically significant, $\chi_{\left(2\right)}^{2}$ = 1.07, *p* adj = 0.368.

**Figure 4:**
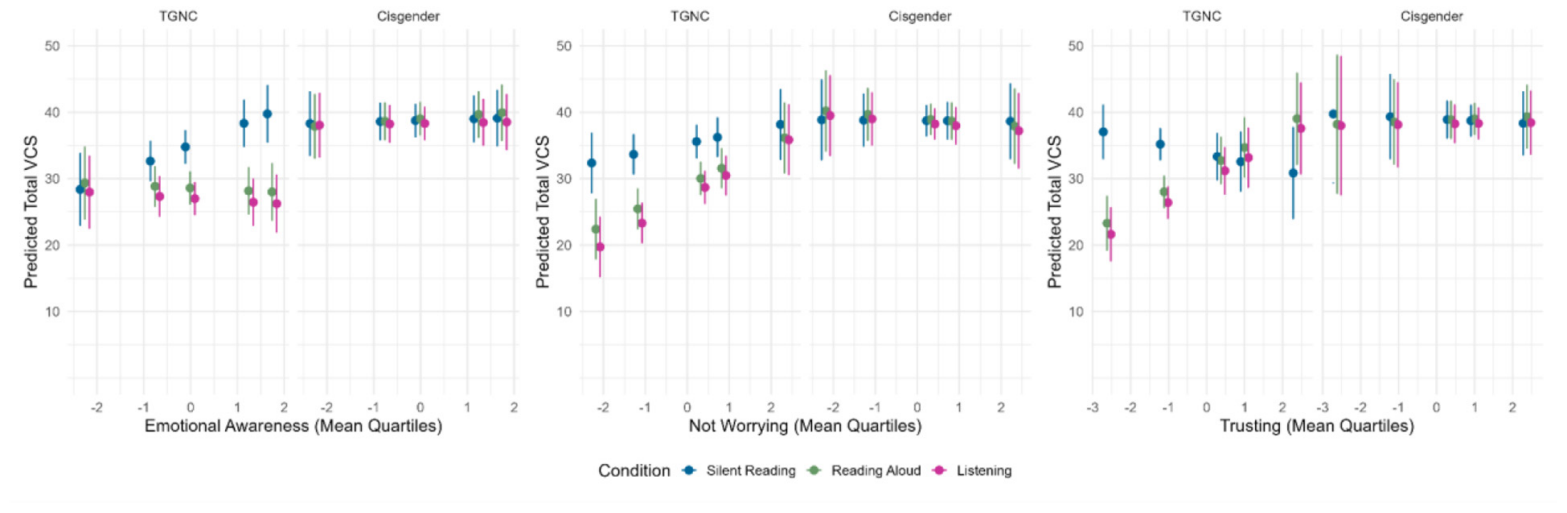
Predicted total VCS scores by emotional awareness, not worrying, and trusting quartiles, condition, and group. Predicted values from a linear model including emotional awareness, not worrying, and trusting (mean-centered), condition (silent reading, reading aloud, listening), and group (TGNC vs. Cisgender), as well as their interactions. Error bars represent 95% confidence intervals. Covariates were divided into quartiles for visualization purposes.

Interoceptive sensibility and its relation with vocal congruence seems to be mostly intertwined with sensory and auditorily feedback. Indeed, we found that, only for TGNC participants, the capability of not worrying about physical discomfort and to trust their own body is associated with higher perceived vocal congruence. Finally, we also found that being able to associate physical sensations to one's own specific feelings or emotions enhanced vocal congruence in the Silent Reading condition for TGNC participants but not for cisgender participants (see Figure 4).

#### 4.3.2 Alexithymia

We investigated differences between the two groups in terms of alexithymia. First, we considered the global scores of the TAS-20 questionnaire. There was a difference between the two groups, *F*_(1)_ = 10.87, *p* = 0.002, such that the mean outcome for the TGNC group (*M* = 56.40; *SD* = 11.60) was significantly higher than that for the cisgender group (*M* = 45.18; *SD* = 10.97) by an average of 11.23 units. So, overall, TGNC participants reported higher levels of alexithymia than cisgender participants. Looking into the subscales of the TAS-20 questionnaire, we found that the groups differed in terms of Difficulty in Identifying Feelings, *F*_(1)_ = 19.61, *p* < 0.001, Difficulty in Describing Feelings (DDF), *F*_(1)_ = 5.469, *p* = 0.024, but not in terms of Externally-Oriented Thinking, *F*_(1)_ = 0.152, *p* = 0.698.

To control for the effect of alexithymia on vocal congruence we fitted four generalized linear mixed models, inserting Total TAS-20 scores and the sub-dimensions of the questionnaire as covariates in interaction with the predictors Condition and Group. The complete results are available in the Supplementary materials.

##### 4.3.2.1 Total TAS-20

**Figure 5:**
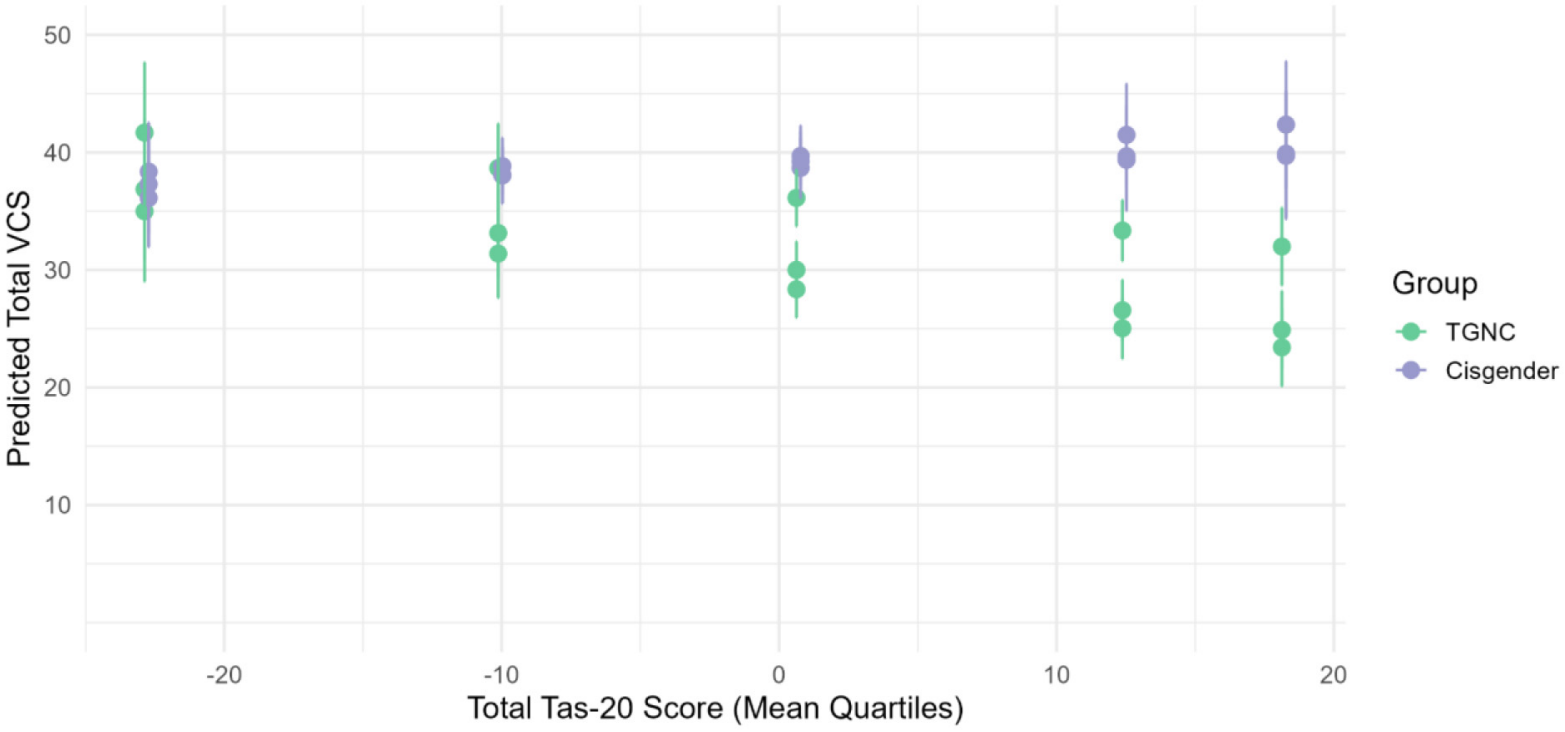
Predicted total VCS scores by total Tas-20 scores quartiles, condition, and group. Predicted values from a linear model including total Tas-20 (mean-centered), condition (silent reading, reading aloud, listening), and group (TGNC vs. Cisgender), as well as their interactions. Error bars represent 95% confidence intervals. Total Tas-20 was divided into quartiles for visualization purposes.

The three-way interaction between Condition, Group, and Total Tas-20 scores did not reach significance, $\chi_{\left(1\right)}^{2}$ = 0.43, *p* = 0.805. However, the model yielded instead a significant two-way interaction between Condition and Group, $\chi_{\left(2\right)}^{2}$ = 38.20, *p* < 0.001, and between Group and Total Tas-20 scores, $\chi_{\left(1\right)}^{2}$ = 6.99, *p* = 0.008. Estimated linear trends show that for the TGNC Group lower scores on the VCS are associated with significantly higher Total Tas-20 scores (β *TGNC* = −0.270, *SE* = 0.09, *t* = −2.946, LCL = −0.45, UCL = −0.08, *p* = 0.005). No other comparisons reached significance, all *ps* > 0.398 (see Figure 5).

The results also indicated a significant main effect of Group, $\chi_{\left(1\right)}^{2}$ = 20.43, *p* < 0.001, and Condition, $\chi_{\left(2\right)}^{2}$ = 58.69, *p* < 0.001. The effect of Total Alexithymia was not significant, $\chi_{\left(1\right)}^{2}$ = 1.97, *p* = 0.160.

##### 4.3.2.2 Externally oriented thinking

The results indicated a three-way interaction between Group, Condition, and EOT, $\chi_{\left(2\right)}^{2}$ = 7.73, *p* adj = 0.034. Estimated trends show that for the TGNC Group lower VCS scores in the Silent Reading Condition are associated with significantly high scores of Externally-Oriented Thinking (β *TGNC* = −0.64, *SE* = 0.30, *t* = −2.126, LCL = −1.24, UCL = −0.03, *p* = 0.038). No other significant comparisons reached significance, all *ps* > 0.193.

The two way interactions between EOT and Condition (*p* adj = 0.487) and Group (*p* adj = 1.75) were not significant. However, a significant interaction between Condition and Group was observed, $\chi_{\left(2\right)}^{2}$ = 63.70, *p* adj < 0.001. We observed significant main effects of Group $\chi_{\left(1\right)}^{2}$ = 28.61, *p* adj < 0.001, and Condition, $\chi_{\left(2\right)}^{2}$ = 72.26, *p* adj < 0.001. The effect of EOT was statistically negligible, $\chi_{\left(1\right)}^{2}$ = 0.001, *p* = 0.970.

Overall, we observed a difference between the two groups in terms of the total alexithymia levels—such that TGNC participants scored significantly higher than cisgender participants. This difference was relevant with respect to how easily they could focus their attention on external stimuli, but critically only in the Silent Reading condition. Specifically, the more TGNC participants oriented their thoughts outward during the Silent Reading, the more incongruent the experience of their inner voice might have been perceived.

#### 4.3.3 Gender identity

Results from the model featuring scores of the MULTI-GIQ suggested there was no relation between gender identity aspects as measured by the questionnaire and vocal congruence scores, all *ps* ≥ 0.057.

#### 4.3.4 Gender minority stress and discrimination

##### 4.3.4.1 Discrimination

We found significant interaction between Condition and Discrimination, $\chi_{\left(1\right)}^{2}$ = 19.37, *p* adj < 0.001. Estimated marginal trends showed that higher scores of Discrimination correspond to lower scores on the VCS in the Silent Reading condition (β *Silent* = −1.44, *SE* = 0.60, *t* = −2.369, LCL = −2.69, UCL = −0.19, *p* = 0.025). No other significant comparison emerged, all *ps* ≥ 0.734.

The model also yielded a significant main effect of Condition, $\chi_{\left(1\right)}^{2}$ = 92.91, *p* adj < 0.001, while there was no main effect of Discrimination, $\chi_{\left(1\right)}^{2}$ = 0.62, *p* adj = 0.415.

##### 4.3.4.2 Internalized transphobia

We found a significant two-way interaction between Condition and Internalized Transphobia, $\chi_{\left(1\right)}^{2}$ = 8.98, *p* adj = 0.018. Estimated marginal trends showed that for TGNC participants lower scores on the VCS scores in the Listening condition are associated with significantly higher scores of Internalized Transphobia (β *Listening* = −0.338, *SE* = 0.15, *t* = −2.142, LCL = −0.66, UCL = −0.01, *p* = 0.041). No other significant comparisons emerged, all *ps* ≥ 0.155.

The model also yielded a significant main effect of Condition, $\chi_{\left(1\right)}^{2}$ = 89.16, *p* adj = < 0.001 and. The main effect of Internalized Transphobia was not statistically significant, $\chi_{\left(1\right)}^{2}$ = 1.83, *p* adj = 0.244.

##### 4.3.4.3 Non-disclosure

We found a significant two-way interaction between Condition and Non-disclosure, $\chi_{\left(2\right)}^{2}$ = 29.18, *p* adj < 0.001. Estimated linear trends showed that for TGNC participants lower scores on the VCS scale in the Reading Aloud condition are associated with significantly high scores of Non-disclosure (β *Reading Aloud* = −0.47, *SE* = 0.22, *t* = −2.088, LCL = −0.93, UCL = −0.008, *p* = 0.046). Also, we found that for TGNC Group lower scores on the VCS scale in the Listening condition are associated with significantly high scores of Non-disclosure (β *Listening* = −0.53, *SE* = 0.22, *t* = −2.346, LCL = −0.99, UCL = −0.06, *p* = 0.026). No other significant comparison emerged, all *ps* ≥ 0.401.

The model also yielded a significant main effect of Condition, $\chi_{\left(2\right)}^{2}$ = 96.45, *p* adj < 0.001. The effect of Non-disclosure was not statistically significant, $\chi_{\left(1\right)}^{2}$ = 1.66, *p* adj = 0.253.

##### 4.3.4.4 Pride

We found a significant two-way interaction between Condition and Pride, $\chi_{\left(1\right)}^{2}$ = 10.75, *p* adj = 0.008. However, no significant relation emerged from *post-hoc* comparisons, all *ps* ≥ 0.217 (β *Silent Reading* = −0.23, *SE* = 0.18, *t* = −1.263, LCL = −0.60, UCL = 0.14, *p* = 0.217; β *Reading Aloud* = 0.06, *SE* = 0.18, *t* = 0.332, LCL = −0.31, UCL = 0.43, *p* = 0.742; β *Listening* = 0.14, *SE* = 0.18, *t* = 0.781, LCL = −0.23, UCL = 0.51, *p* = 0.441).

The model also yielded a significant main effect of Condition, $\chi_{\left(1\right)}^{2}$ = 89.80, *p* adj < 0.001. The effect of Pride was not statistically significant, $\chi_{\left(1\right)}^{2}$ = 0.003, *p* adj = 0.957.

##### 4.3.4.5 Community connectedness

We found a significant two-way interaction between Condition and Community Connectedness, $\chi_{\left(2\right)}^{2}$ = 7.32, *p* adj = 0.038. However, no significant relation emerged from *post-hoc* comparisons, all *ps* ≥ 0.140 (β *Silent Reading* = −0.48, *SE* = 0.31, *t* = −1.519, *p* = 0.140, LCL = −1.13, UCL = 1.69; β *Reading Aloud* = 0.01, *SE* = 0.31, *t* = 0.062, LCL = −0.63, UCL = 0.67, *p* = 0.950; β *Listening* = 0.002, *SE* = 0.31, *t* = 0.008, LCL = −0.64, UCL = 0.65, *p* = 0.996).

The model also yielded a significant main effect of Condition, $\chi_{\left(2\right)}^{2}$ = 88.56, *p* adj < 0.001. The effect of Community Connectedness was not statistically significant, $\chi_{\left(1\right)}^{2}$ = 0.27, *p* adj = 0.676.

Altogether, the results from the questionnaire addressing factors related to stress and discrimination driven by gender identity provide a nuanced picture of their relation with vocal congruence. Indeed, we found that TGNC participants who had experienced more social discrimination on the basis of their gender identity also reported lower vocal congruence in the Silent Reading condition—suggesting these negative experiences might have been systematically internalized. By contrast, TGNC participants who internalized social prejudice about TGNC identity and were less confident in self-disclosure do not recognize their outer voice (Reading Aloud and Listening), reporting lower congruence scores.

## 5 Discussion

In this study, we explored vocal congruence across different gender identities, comparing cisgender with transgender and gender nonconforming participants (TGNC) on a self-voice perception task in three conditions: (i) Silent Reading, (ii) Reading Aloud, and (iii) Listening to their recorded voice. We then investigated whether the observed differences could be accounted for by individual differences in interoceptive sensibility—the awareness of internal bodily sensations—and alexithymia. Additionally, we explored whether these effects were shaped by the flexibility of participants' gender identity representations and influenced by experiences of gender-related discrimination.

We predicted that TGNC participants would report lower scores of vocal congruence in conditions in which they were asked to pay attention to their outer voice compared to cisgender participants—while reporting higher congruence following silent reading. Also, we expected that reading a gender-stereotyped text would decrease vocal congruence in TGNC participants. Results partially confirmed our first prediction, with TGNC participants experiencing lower vocal congruence in both Reading Aloud and Listening conditions compared to cisgender participants. Unexpectedly, this difference persisted in the Silent Reading condition. Results from the Inner-Outer Voice Congruence Rating consolidated these findings, showing that TGNC participants perceived a greater mismatch between their internal representation of their inner and outer voice than cisgender participants.

Taken together, the results from the main tasks specifically addressing vocal congruence underscore the dynamic and reciprocal relationship between core components of one's self-concept, such as gender identity, and more embodied identity characteristics, such as the voice. Importantly, our data suggest that for individuals whose gender identity does not fit within binary, heteronormative categories, this reciprocal loop may also be a source of difficulty. Notably, although one might have expected TGNC participants to experience greater vocal incongruence only when directly confronted with their own voice—whether recorded or not—such mismatch also emerged during internal reading. This pattern may point to subtle forms of internalized stigma, whereby even private aspects of experience, such as one's inner voice, are perceived as misaligned with the voice participants would accept and desire.

Our second prediction concerned the impact of gender stereotypes on the perception of vocal congruence in relation to gender identity. No significant differences emerged between the two groups as to the type of text (gendered vs. neutral), with TGNC participants consistently exhibiting higher vocal incongruence, regardless of the gendered content of the texts. It is possible that the absence of an effect is related to the task instructions. Participants were neither required to memorize the content of the text nor to convey it to another interlocutor. Thus, we might hypothesize that the semantics of the text could be overridden by self-related cognitive, emotional, and interoceptive variables associated with the reading process itself, whether it is internal or external. We further addressed the role of interoceptive sensibility in voice perception within different gender identities. Previous studies have demonstrated that interoception—particularly through bone conduction and vibrotactile sensations—can influence self-voice perception (Crow et al., 2021; Orepic et al., 2022; Smeltzer et al., 2023). Overall, TGNC participants reported lower ability to self-regulate internal states and lower trust in bodily signals compared to cisgender participants. In relation to vocal congruence, TGNC participants who showed greater Emotional Awareness also tended to report a stronger sense of congruence in the Silent Reading condition. No similar relationship was observed for cisgender participants. We speculate that Emotional Awareness may enhance vocal congruence only in the Silent reading condition, but not in the Reading Aloud and Listening conditions—where heightened distress may override the protective effects of Emotional Awareness. Furthermore, visual inspection of the data suggests that as Emotional Awareness increases, vocal congruence improves more markedly in the Silent Reading condition than in the Reading Aloud and Listening conditions, indicating a heightened sensitivity to social exposure. This pattern may highlight a potential distinction between private self-perception and socially mediated voice experiences.

Interestingly, for the TGNC group (but not for cisgender participants) the tendency not to experience emotional distress with sensations of physical discomfort was associated with higher VCS scores during both the Reading Aloud and Listening conditions. This suggests that being able to self-regulate one's own emotional distress when faced with a potentially misaligned auditory stimulus (i.e., participants' voice perceived as incongruent) might have acted as a protective factor during the task.

Additionally, higher trust in bodily signals was associated with higher vocal congruence among TGNC individuals in both the Listening and Reading Aloud conditions. We can speculate that experiencing one's own body sensations as safe and trustworthy again acts as a protection against both psychological and physical discomfort arising from the perception of vocal incongruence. Visual inspection of the data further suggests that, at higher levels of trust in bodily signals, vocal congruence tends to align across inner (Silent Reading) and outer (Reading Aloud and Listening) voice conditions.

In our study we found that higher overall alexithymia was associated with lower vocal congruence within the TGNC participants, regardless of the experimental condition. This result highlights a general role of emotional awareness in shaping the perception of vocal congruence within this population. Alexithymia is currently understood as a multifaceted and dimensional construct (Bagby et al., 2020). According to some proposals, the ability to identify and express emotions might may be influenced, among other factors, by gender socialization, with men and women being guided to rely on different cues when gauging their internal states (Berke et al., 2018; Pennebaker and Roberts, 1992). Male participants usually score higher than female participants in interoceptive accuracy tasks, as revealed by heartbeat counting tasks (for a review, see: Prentice and Murphy, 2022; see also Desmedt et al., 2020). On the other hand, women outperform men in emotion recognition and emotional awareness (Thompson and Voyer, 2014), while men consistently report higher levels of alexithymia compared to women (Levant et al., 2009). However, no conclusive evidence on the presence of differences in interoceptive and emotional abilities between genders exists, and different models of explanations have been proposed.

Interestingly, for TGNC participants, also higher externally-oriented thinking (tendency to focus on concrete, practical, and objective aspects of the external environment, rather than on internal contents; Luminet et al., 2004; Lumley and Bazydlo, 2000), was related to reduced vocal congruence, but only during the Silent Reading condition. This suggests that TGNC participants with high Externally-Oriented Thinking specifically struggle more to focus on the internal voice during the Silent Reading condition as compared to the Reading Aloud and Listening conditions. Visual inspection of the data further suggests that, at higher levels of External-Oriented Thinking, the outward focus on external stimuli may distract from internal signals, leading to a more “fragmented” experience of inner voice, while articulatory and auditory feedback would instead contribute to the grounding of bodily self. In light of these results, we believe future research is needed to further investigate prevalence and impact of alexithymia within the TGNC population.

Overall there was no effect of participants ontological beliefs about gender/sex on their perception of vocal congruence. However, it seemed vocal congruence judgments of TGNC participants were partly modulated by gender-related discrimination and minority stress levels. We found both proximal (i.e., internalized transphobia and non-disclosure) and distal (i.e., discrimination) stressors to impact vocal congruence perception among different experimental conditions. Distal stressors, which are more related to structural stigma (i.e., social conditions, cultural norms and institutional policies that restrict opportunities and resources, as well as wellbeing for stigmatized groups, see Hatzenbuehler et al., 2024) negatively affected the inner perception of vocal congruence, with higher reported experience of discrimination associated with lower scores in the Silent Reading condition. Remarkably, this finding might help elucidate why, contrary to our initial expectation, we also found cisgender and TGNC participants to differ in their vocal congruence judgments on the main task also in the inner reading condition. Conversely, proximal stressors like internalized transphobia and more crucially the tendency not to disclose one's own gender identity—for example by modifying the way of speaking—were both associated with lower VCS scores in Listening and both Reading Aloud and Listening, respectively.

To our knowledge, this is the first study to directly compare vocal congruence between two balanced groups of cisgender and TGNC participants. Our findings are in line with previous literature on voice perception in TGNC individuals, adding layers to this literature by tackling the unexplored topic of vocal congruence in this population. However, this study has some methodological limitations that are worth mentioning. First, we did not control for levels of vocal discomfort prior to the experimental procedure. Second, we are aware that the TGNC group might collapse very different experiences regarding GAHT (Gender-Affirming Hormone Therapy) status or social transition. However, following the request of the center's operators we deliberately refrained from asking for more detailed personal data, besides the eventual stage of gender-affirming procedures and voice training interventions. This was motivated by the necessity of limiting participants' potential discomfort experiences, but we acknowledge future research might delve deeper into further factors that may influence this population's vocal congruence. In addition, the specific selection of linguistic stimuli may have limited the influence of gender primes on VCS scores. We also used the original version of the MAIA scale (Mehling et al., 2012), which, while widely used, has since been revised to improve psychometric properties and address conceptual overlaps across subscales. Lastly, the difficulty in recruiting TGNC participants led to a relatively small sample size, which limited the generalizability of the findings and prevented comparisons across different gender identities within each group, including as a function of the gendered content of the texts. Future research should aim to address these limitations to better capture the complex relationship between vocal congruence, language, and gender identity.

## 6 Conclusion

This study investigates vocal congruence in two groups that differ in their gender identity. We found that transgender and gender non-conforming participants experienced lower vocal congruence compared to cisgender participants in all experimental conditions of the vocal congruence task, with a larger difference observed when an external auditory feedback was present. This experience of incongruence appears to be modulated by interoceptive sensibility and levels of alexithymia in transgender and gender non-conforming participants but not in cisgender ones. In addition, minority-stress related factors were also associated with higher perceived vocal incongruence in the TGNC group. Further research is needed to deepen our understanding of the relationship between inner experiences and voice perception and to clarify the reciprocal relationship between self-identity and self-voice perception.

## Data Availability

The datasets presented in this study can be found in online repositories. The names of the repository/repositories and accession number(s) can be found at: https://osf.io/v2gtn/.
