# Supplementary material for: Gender identity impacts the perception of vocal congruence
Source: Front Cognit. 2026 Mar 10;5:1638501. doi: 10.3389/fcogn.2026.1638501 (PMC13281043; doi:10.3389/fcogn.2026.1638501)
Supplement: Supplementary file 1 [file Data_Sheet_1.docx]

# **Supplementary Materials**

**Materials**

**Table 1.** *Materials for the Vocal Congruence Task.*

| **Original Italian Version** | **English Translation** | **Reference** |
| --- | --- | --- |
| Il luogo nel quale si fanno le compere giornaliere in ogni città greca è la «piazza», detta agorà, che non è soltanto la piazza del mercato, ma il maggior ritrovo della vita cittadina sino alle prime ore del pomeriggio. In generale per le vie della città c’è poca gente. Le vie della città sono strette, sono fiancheggiate da muri nudi e disadorni, di solito senza finestre, o con qualche finestrina in alto; manca la varietà e l’attrattiva dei grandi magazzini; le botteghe sono bugigattoli, e, per lunghi tratti, si rasenta l’esterno di case impenetrabili, chiuse e tristi come prigioni. Nelle ore intorno al mezzogiorno, quel periodo della giornata che i Greci chiamavano appunto «il mercato pieno», tutti i cittadini si riversavano nella grande piazza. Questa è la ragione per la quale, leggendo i testi greci, si ha l’impressione che, anche nelle città più grandi, i cittadini, fra loro, si conoscessero un po’ tutti." | The place where daily shopping is done in every Greek city is the "square," called the agora, which is not only the marketplace but also the main gathering spot of city life until the early afternoon. In general, the streets of the city are sparsely populated. The city streets are narrow and lined with bare, undecorated walls, usually without windows or with only a small window high up; they lack the variety and appeal of large department stores. The shops are small, cramped spaces, and for long stretches, one walks past the exterior of impenetrable houses—closed and somber like prisons. Around midday—what the Greeks called “the full market”—all the citizens would pour into the large square. This is why, when reading Greek texts, one gets the impression that, even in the largest cities, the citizens all more or less knew one another. | adapted from Paoli, U. E. (1957). Come vivevano i Greci. Edizioni Radio Italiana. |
| Ancor prima che i bambini nascano, molti genitori tendono ad avere un diverso atteggiamento rispetto al sesso. Se si pensa che sarà un maschio lo si immagina portato per le attività sportive, teso al successo, forte e indipendente. La maggior parte dei giocattoli in commercio, di conseguenza, non è concepita come libero stimolo alla fantasia e alla creatività infantile, ma è offerta in vista delle diverse aspettative degli adulti relative ai maschi e alle femmine. Tra i giocattoli per maschi dominano oggetti ricchi di informazioni sugli aspetti geometrici e fisici del mondo e sulle regole che definiscono le possibilità aggregative dei vari pezzi. Molto diffusi, poi, sono giocattoli in vario modo ispirati alla guerra, o ad eroi superpotenti, o a prove iniziatiche. Valorizzano la competizione: avere il controllo, avere il potere, vincere, conquistare. Accanto a ‘maschio’ troviamo sul dizionario italiano: che ha del forte; cuore maschio, virtù maschia; stile maschio, architettura maschia = grandiosa. | Even before children are born, many parents tend to adopt different attitudes based on the expected sex of the child. If they believe it will be a boy, they often imagine him as athletic, success-driven, strong, and independent. As a result, most toys on the market are not designed simply to stimulate children's imagination and creativity freely, but are instead offered in line with adults’ differing expectations for boys and girls. Among boys' toys, those that dominate are rich in information about geometric and physical aspects of the world, as well as about the rules that govern how different pieces can be assembled. Also widespread are toys inspired in various ways by war, superpowered heroes, or initiation challenges. These toys emphasize competition: gaining control, having power, winning, conquering. In the Italian dictionary, next to the word maschio (male), we find: "associated with strength"; cuore maschio (brave heart), virtù maschia (manly virtue); stile maschio, architettura maschia—meaning grand or imposing. | adapted from Priulla, G., Banci, M., and Sammartino, G. (2020). L’abbecedario degli stereotipi di genere. |
| Era una modesta casa di periferia che aveva però l'innegabile pregio di essere poco distante dal mare; per questo potevo vedere l'andirivieni delle navi e la luce del faro mi faceva compagnia dall'imbrunire all'alba. Il viaggio in bicicletta fino alla punta del promontorio durava all'incirca un quarto d'ora e offriva la vista di quel miscuglio di meraviglie e miserie che sono le città portuali di tutto il mondo, ma ancora di più quelle della costa mediterranea dell'Africa. Dalla parte opposta a quella da cui arrivavo c’era la distesa delle raffinerie, delle fabbriche, gli impianti per il gas, il porto, ma non mi avventuravo mai fin là con la mia piccola bicicletta, non avrei saputo che farci e poi m’incuteva anche un po’ di paura. Dopo la visita al faro, riprendevo la via di casa: man mano che mi avvicinavo al nostro sobborgo, le costruzioni apparivano più povere e le rare automobili erano sgangherate. | It was a modest house in the suburbs, but it had the undeniable advantage of being close to the sea; because of that, I could watch the comings and goings of ships, and the lighthouse beam kept me company from dusk until dawn. The bike ride to the tip of the promontory took about fifteen minutes and offered a view of that mixture of wonders and hardships typical of port cities all over the world—though even more so in those along the Mediterranean coast of Africa. On the opposite side from where I approached was a sprawl of refineries, factories, gas plants, and the port itself. But I never ventured that far on my little bicycle; I wouldn't have known what to do there, and to be honest, it frightened me a bit. After visiting the lighthouse, I would head back home: as I got closer to our neighborhood, the buildings looked poorer, and the few cars I passed were ramshackle. | adapted from Rava, C. (2012). Un mare di silenzio. Garzanti. |
| Ancor prima che i bambini nascano, molti genitori tendono ad avere un diverso atteggiamento rispetto al sesso. Se si pensa che sarà una femmina la si immagina gentile, sensibile, portata per le relazioni e la vita matrimoniale. La maggior parte dei giocattoli in commercio, di conseguenza, non è concepita come libero stimolo alla fantasia e alla creatività infantile, ma è offerta in vista delle diverse aspettative degli adulti relative ai maschi e alle femmine. Tra i giocattoli per femmine dominano due grandi categorie: bellezza e seduttività (trucco, parrucchiera, moda, ballo) e lavori di cura (cucina, casa, lavoro domestico, oppure maternità). Molto diffusi, poi, sono gli oggetti in vario modo ispirati alle relazioni e capaci di indurre un attaccamento emotivo, come le bambole. Valorizzano la capacità di interagire e costruire relazioni: essere concilianti, disponibili, dolci ed esteticamente gradevoli. Sul dizionario, sinonimi che possono essere usati al posto di ‘femminile’ sono: debole, fiacco, molle, delicato, fragile. | Even before children are born, many parents tend to adopt different attitudes based on the child's sex. If they believe it will be a girl, she is often imagined as gentle, sensitive, and naturally inclined toward relationships and married life. As a result, most toys on the market are not designed simply to stimulate children's imagination and creativity freely, but are instead shaped by adults’ differing expectations for boys and girls. Among toys marketed to girls, two main categories dominate: beauty and seductiveness (makeup, hairstyling, fashion, dance), and caregiving roles (kitchen sets, housekeeping, domestic chores, or motherhood). Also widespread are toys inspired in various ways by relationships and capable of fostering emotional attachment, such as dolls. These toys emphasize skills related to interaction and relationship-building: being conciliatory, available, sweet, and aesthetically pleasing. In the dictionary, synonyms often listed alongside feminine include: weak, limp, soft, delicate, fragile. | adapted from Priulla, G., Banci, M., and Sammartino, G. (2020). L’abbecedario degli stereotipi di genere. |

**Analysis of VCS Total Distribution**

The distribution of VCS total scores was assessed using visual inspection of histograms and Q–Q plots alongside formal normality tests. The distribution appeared approximately unimodal and bell-shaped, with observations primarily concentrated in the mid-range (see Fig. S1).


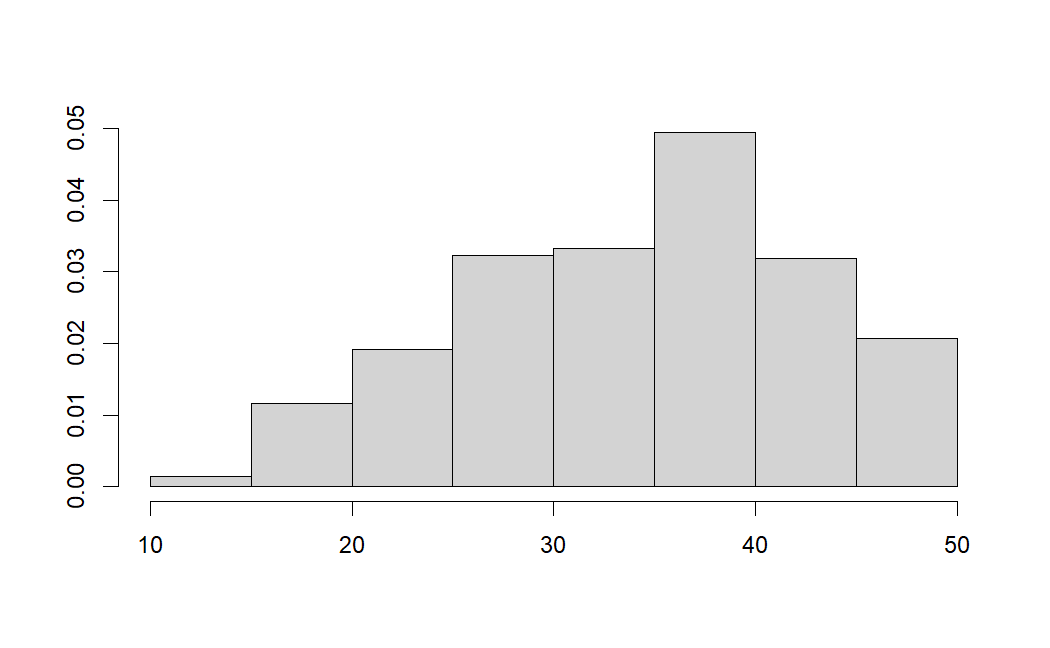


**[Fig S1. *Distribution of VCS Total Scores*]**The histogram displays the density distribution for the sample (*n* = 44).

While the Shapiro-Wilk test (via “*stats*” R’ Package) indicated a statistically significant deviation from normality (W = 0.9746, *p* < .001), visual diagnostics revealed that this deviation was primarily confined to the extreme tails, reflecting the bounded nature of the 10–50 sum score (see Fig. S2).

**
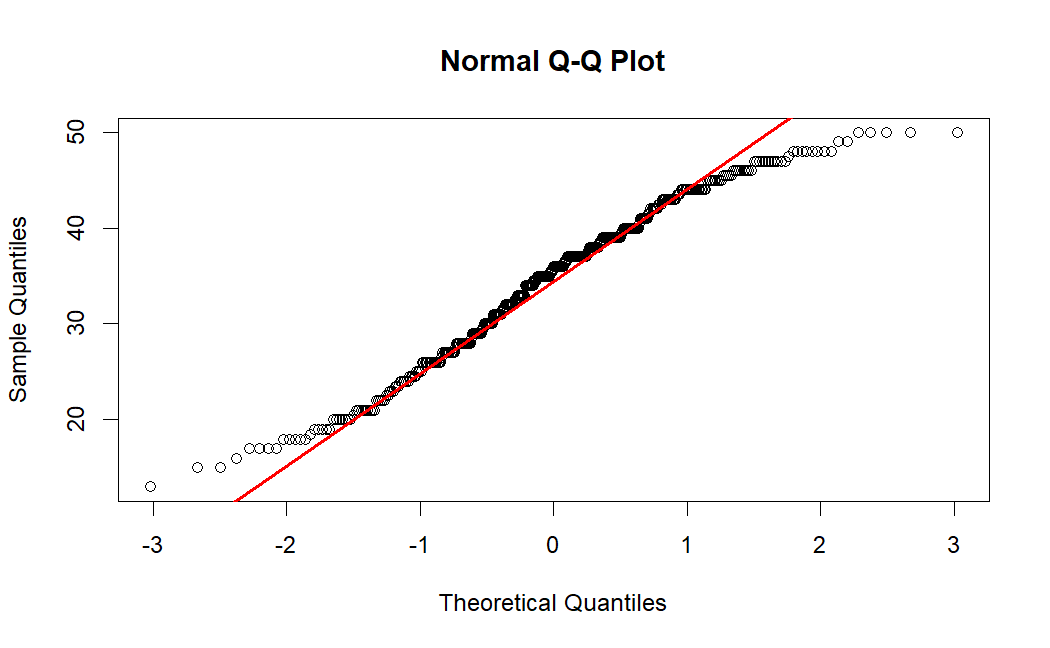
**

### **[Fig S2. *Normal Q–Q Plot of VCS Total Scores*]** Theoretical quantiles are plotted against observed sample quantiles. The solid red line represents the expected pattern if the data were perfectly normally distributed.

### **Model Diagnostic for the main VCS Total model**

### Assumptions for the linear mixed-effects model were verified through visual and formal diagnostics (Fig. S3). Validation using simulated scaled residuals via the “*DHARMa”* package confirmed an appropriate fit, with no significant evidence of Kolmogorov-Smirnov deviations (*p* = .270), outliers (*p* = .564), or overdispersion (*p* = .824; Fig. S4). Minor quantile deviations observed in the residual-versus-predicted plots were deemed non-problematic as the combined adjusted quantile test remained non-significant.

### **
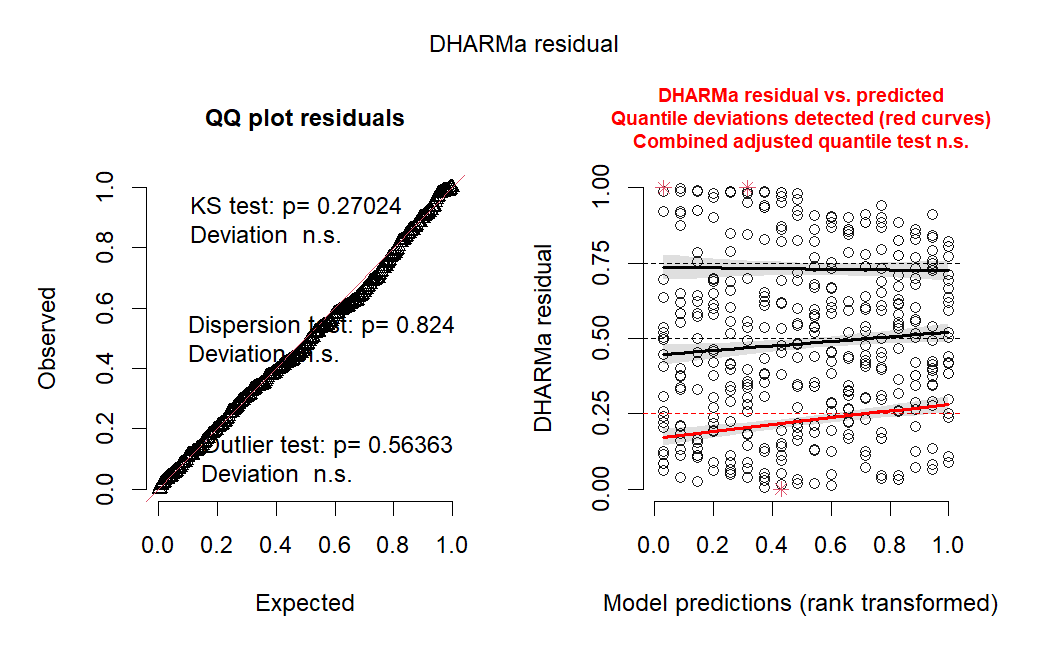
**

### **[Fig S3. *DHARMa Residual Diagnostics for the Linear Mixed-Effects Model*]** The left panel shows a Q–Q plot of scaled residuals with a non-significant Kolmogorov-Smirnov (KS) test (*p* = .270), indicating no major distributional deviations. The right panel displays residuals against rank-transformed model predictions; although minor quantile deviations were detected at the lower end (red lines), the combined adjusted quantile test was non-significant, confirming adequate model specification.


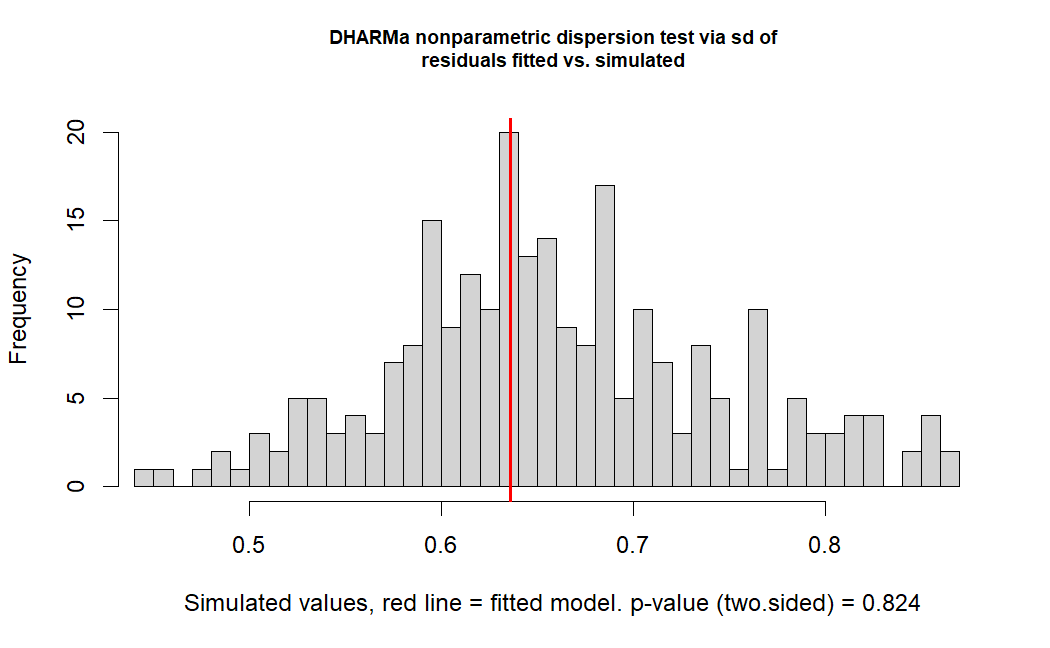


### **[Fig S4. *DHARMa Dispersion Test*]** The histogram displays the distribution of simulated standard deviations of residuals compared to the observed value (indicated by the red vertical line).

###

###

###

###

### **Supplementary Results - VCS Items**

Even though the validation study of the VCS scale (Crow et al., 2021) did not report distinct subscales, we were interested in possible differences between the groups over the different facets of vocal congruence assessed through this scale. We first visually inspected the density distributions of the two Groups for each of the 10 items (see Fig. S5).


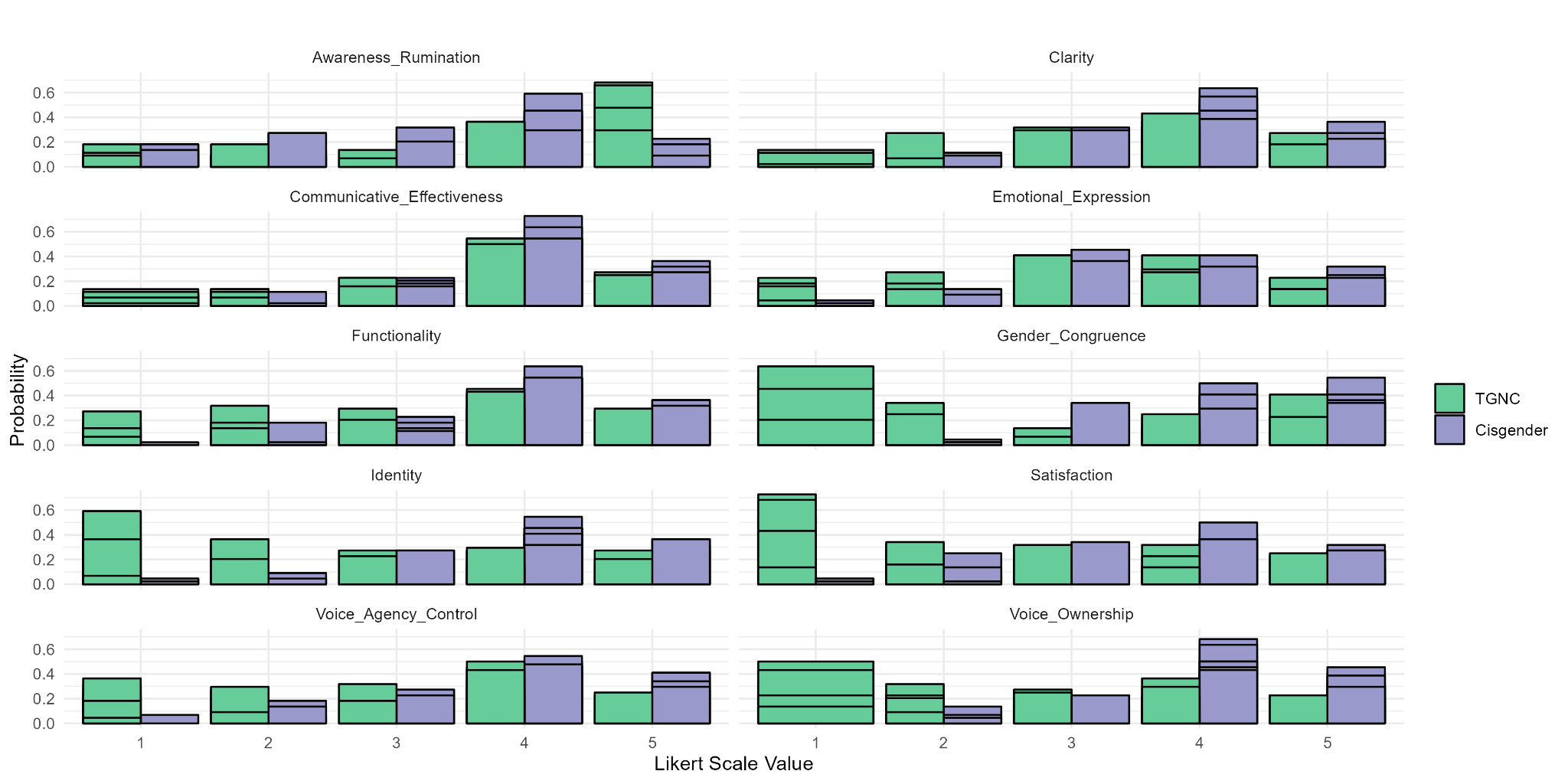


**[Fig S5. *Distribution of Responses across the 10-items of the VCS scale by Groups*]**This figure presents Probability distributions that show response patterns for Cisgender and TGNC participants across ten voice-related dimensions of the VCS scale measured on 5-point Likert scales (1 = *strongly disagree*; 5 = *strongly agree).*

Wilcoxon rank sum test suggests that the two groups significantly differ along all the 10 subdimensions, as reported in Table S1.

**Table S1. Wilcoxon Rank Sum Test Results by Groups (TGNC vs. Cisgender) on VCS Items**

| ***VCS Item*** | ***W*** | ***p-value*** | ***Significance*** |
| --- | --- | --- | --- |
| Voice Ownership | 52618 | < .001 | *** |
| Functionality | 45961 | < .001 | *** |
| Identity | 55428 | < .001 | *** |
| Voice Agency & Control | 48078 | < .001 | *** |
| Satisfaction | 53220 | < .001 | *** |
| Awareness / Rumination | 21564 | < .001 | *** |
| Clarity | 43018 | < .001 | *** |
| Communicative Effectiveness | 40861 | < .001 | *** |
| Emotional Expression | 45146 | < .001 | *** |
| Gender Congruence | 57529 | < .001 | *** |

***Note***. Significance levels: *** p < .001. Tests are Wilcoxon rank sum with continuity correction.

**Supplementary Results - Covariates**

### **Multidimensional Assessment of Interoceptive Awareness Questionnaire (MAIA)**

No significant differences were found between TGNC and cisgender participants across multiple measures. Specifically, Emotional Awareness scores did not differ between the groups, F(1) = 0.09, p = .764, with TGNC participants scoring M = 4.31 (SD = 1.18) and cisgender participants scoring M = 4.20 (SD = 1.31). Similarly, Noticing scores showed no difference, F(1) = 0.77, p = .385, with cisgender participants scoring M = 4.75 (SD = 0.79) and TGNC participants M = 4.47 (SD = 1.21). Attention Regulation scores were also comparable, F(1) = 1.80, p = .186, with cisgender participants reporting M = 3.77 (SD = 1.13) and TGNC participants M = 3.29 (SD = 1.22). For Not Distracting, the groups did not differ significantly, F(1) = 1.03, p = .316, with means of M = 3.45 (SD = 0.95) for cisgender and M = 3.15 (SD = 0.98) for TGNC participants. Similarly, Not Worrying scores showed no significant group difference, F(1) = 0.95, p = .334, with cisgender participants scoring M = 3.34 (SD = 0.95) and TGNC participants M = 3.02 (SD = 1.19). Lastly, Body Listening approached but did not reach significance, F(1) = 3.52, p = .067, with cisgender participants scoring M = 3.86 (SD = 1.13) and TGNC participants M = 3.18 (SD = 1.26).

**Noticing.** The three-way interaction between Condition, Group, and Noticing was not significant, *χ2*(2) = 0.26, *p adj* = 0.759, as well as the two-way interactions involving Noticing (all *ps* > .309). The model yielded a significant interaction between Condition and Group, *χ2*(2) = 64.00, *p adj* < .001, and significant main effects of Condition, *χ2*(2) = 68.70, *p adj* < .001, and Group, *χ2*(1) = 29.11, *p adj* < .001.

**Attention Regulation.**  The three-way interaction between Group, Condition, and Attention Regulation did not reach the standard threshold for significance, *χ2*(2) = 4.83, *p adj* = .133, as well as the two-way interaction between Condition and Attention Regulation (*p adj* = .059), and between Group and Attention Regulation (*p adj* = .721). We observed a significant interaction between Condition and Group, *χ2*(2) = 52.83, *p adj* < .001, and significant main effects of Condition, *χ2*(2) = 60.09, *p adj* < .001, and Group, *χ2*(1) = 24.53, *p adj* < .001. We found no significant main effect of Attention Regulation, *χ2*(1) = 2.90, *p* *adj*= .133.

**Not Distracting.** The three-way interaction between Condition, Group, and Not Distracting was not significant, *χ2*(2) = 2.59, *p adj* = .565, as well as the two-way interactions involving Noticing (*ps* <= .511). The model yielded a significant interaction between Condition and Group, *χ2*(1) = 62.82, *p adj* < .001, and significant main effects of Condition χ2(2) = 71.19, *p adj* < .001, and Group, *χ2*(1) = 33.61, *p adj* < .001. The Not-Distracting covariate was not statistically significant, *χ2*(1) = 4.47, *p adj* = .058.

**Self Regulation.** The three-way interaction between Condition, Group, and Self Regulation did not reach significance, *χ2*(2) = 4.23; *p adj* = .172), as well as the two-way interactions including the covariate (all *ps* >= .356). We found a significant two-way interaction between the Condition and Group, *χ2*(1) = 44.57, *p adj* < .001, as well as significant main effects of Group, *χ2*(1) = 16.55, *p adj* < .001, and Condition, *χ2*(2) = 55.62, *p adj* < .001. The main effect of Self-Regulation was statistically significant, *χ2*(1 ) = 5.26, *p* = .038.

**Body Listening.** The three-way interaction between Condition, Group, and Body Listening was not significant, *χ2*(2) = 1.40, *p adj* = 0.556, as well as the two-way interactions involving Noticing (*ps* <= .368). The model yielded a significant interaction between Condition and Group, *χ2*(2) = 59.45, *p adj* < .001, and significant main effects of Condition, *χ2*(2) = 59.08, *p adj* < .001, and Group, *χ2*(1) = 24.12, *p adj* < .001. The Body Listening covariate was not statistically significant, *χ2*(1) = 0.49, *p adj* = .549.

**Emotion Regulation Questionnaire (ERQ)**

We found no difference between the groups both for Cognitive Reappraisal, *F*(1) = 0.91, *p* = .345, *M* TGNC = 4.35; *SD* = 1.13; *M* cisgender = 4.67; *SD* = 1.07; and Expressive Suppression, *F*(1) = 2.257, *p* = .14, *M* TGNC = 3.78; *SD* = 1.01; *M* cisgender = 3.27; *SD* = 1.23). To control for the effect of Cognitive Reappraisal and Expressive Suppression on vocal congruence we fitted two generalised linear mixed models, inserting the ERQ subscales in interaction terms with the predictors Condition and Group.

**Cognitive Reappraisal.** The three-way interaction between Condition, Group, and Cognitive Reappraisal was not significant, *χ2*(2) = 3.44, *p adj* = 0.286, as well as the two-way interactions involving Cognitive Reappraisal (*ps* <= .520). The model yielded a significant interaction between Condition and Group, *χ2*(2) = 63.39, *p adj* < .001, and significant main effects of Condition, *χ2*(2) = 71.54, *p adj* < .001, and Group, *χ2*(2) = 63.39, *p adj* < .001. The Cognitive Reappraisal covariate was not statistically significant, *χ2*(1) = 3.38, *p adj* = .117.

**Expressive Suppression.** The three-way interaction between Condition, Group, and Expressive Suppression was not significant, *χ2*(2) = 0.23, *p adj* = 0.891, as well as the two-way interactions involving Expressive Suppression (*ps* <= .569). The model yielded a significant interaction between Condition and Group, *χ2*(2) = 55.10, *p adj* < .001, and significant main effects of Condition, *χ2*(2) = 64.76, *p adj* < .001, and Group, *χ2*(1) = 28.53, *p adj* < .001. The Expressive Suppression covariate was not statistically significant, *χ2*(1)= 0.835, *p adj* = .520.

**Toronto Alexithymia Scale (Tas-20)**

**Difficulty Identifying Feeling (DIF).** The three-way interaction between Group, Condition, and DIF was not significant, *χ2*(2) = 0.57, *p adj* = .781. Notably, a significant two-way interaction was found between Condition and DIF, *χ2*(2) = 9.32 *p adj* = .017. However, the two-way interaction between Group and DIF was not significant, *χ2*(2) = 2.70, *p adj* = .142. The model also indicated a significant interaction between Condition and Group *χ2*(2) = 24.04, *p adj* < .001, as well as significant main effects of Condition, *χ2*(2) = 41.857, *p adj* < .001, and Group, *χ2*(1) = 14.64, *p adj* < .001. The effect of DIF was not significant, *χ2*(1) = 1.58, *p adj* < .262.

***Difficulty Describing Feelings (DDF).*** The three-way interaction between Group, Condition, and DDF was not significant, *χ2*(2) = 0.66, *p adj* =.781. Notably, we observed a significant two-way interaction between Group and DDF, *χ2*(1) = 6.34, *p adj* = .020. However, the two-way interaction between Condition and DDF was not significant, *χ2*(2) = 5.294, *p adj* = .106. The model also indicated a significant interaction between Condition and Group, *χ2*(2) = 42.54, *p adj* < .001, as well as significant main effects of Condition, *χ2*(2) = 64.51 *p adj* < .001, and Group, *χ2*(1) = 26.24, *p adj* < .001). The effect of DIF was not significant, *χ2*(1) = 0.679, *p adj* = .487.

**Multi-Gender Identity Questionnaire (Multi-GIQ)**

**Satisfaction with one's own affirmed gender (Woman).** The three-way interaction between Condition, Group, and satisfaction with one's own affirmed gender (woman) was not significant, *χ2*(2) = 0.11, *p adj* < .954, as well as the two-way interactions involving the covariate (all *ps* > .649). The model yielded a significant two-way interaction between Condition and Group, *χ2*(2) = 45.76, *p adj* < .001, as well as significant main effects of Condition, *χ2*(2) = 55.97, *p adj* < .001, and Group, *χ2*(1) = 13.78, *p adj* = .001. However, satisfaction with one's own affirmed gender (woman) showed no significant main effect, *χ2*(1) = 0.767, *p adj* = .623.

**Satisfaction with one's own affirmed gender (Man).** The three-way interaction between Condition, Group, and satisfaction with one's own affirmed gender (man) was not significant, *χ2*(2) = 0.16, *p adj* < .941, as well as the two-way interactions involving the covariate (all *ps* > .745). The model yielded a significant two-way interaction between Condition and Group, *χ2*(2) = 23.31, *p adj* < .001, as well as significant main effects of Condition, *χ2*(2) = 30.32, *p adj* < .001, and Group, *χ2*(1) = 24.58, *p adj* < .001. However, satisfaction with one's own affirmed gender (man) showed no significant main effect, *χ2*(1) = 0.64, *p adj* = .642.

**Wish to be the other gender (Woman).** The three-way interaction between Condition, Group, and wish to be the other gender (woman) was not significant, *χ2*(2) = 0.96, *p adj* < .745, as well as the two-way interactions involving the covariate (all *ps* > .623). The model yielded a significant two-way interaction between Condition and Group, *χ2*(2) = 31.62, *p adj* < .001, as well as significant main effects of Condition, *χ2*(2) = 25.71, *p adj* < .001, and Group, *χ2*(1) = 8.58, *p adj* = .008. However, wishing to be the other gender (woman) showed no significant main effect, *χ2*(1) = 0.469, *p adj* = .679.

**Wish to be the other gender (Man).** The three-way interaction between Condition, Group, and wish to be the other gender (man) was not significant, *χ2*(2) = 0.65, *p adj* < .833. A significant two-way interaction emerged with Group, *χ2*(1) = 6.01, *p adj* = .030. The two-way interaction with Condition was not significant, *χ2*(1) = 1.09, *p adj* = .745. The model yielded a significant two-way interaction between Condition and Group, *χ2*(2) = 25.99, *p adj* < .001, as well as significant main effects of Condition, *χ2*(2) = 46.69, *p adj* < .001, and Group, *χ2*(1) = 21.82, *p adj* < .001. However, wishing to be the other gender (man) showed no significant main effect, *χ2*(1) = 0.445, *p adj* = .683.

**Dislike of one’s own sexed body (Woman).** The three-way interaction between Condition, Group, and dislike of one’s own sexed body (woman) was negligible, *χ2*(2) = 0.92, *p adj* < .750, as well as the two-way interactions involving the covariate (all *ps* > .316). The model yielded a significant two-way interaction between Condition and Group, *χ2*(2) = 19.54, *p adj* < .001, as well as significant main effects of Condition, *χ2*(2) = 14.92, *p adj* = .001, and Group, *χ2*(1) = 8.52, *p adj* = .008. However, dislike of one’s own sexed body (woman) showed no significant main effect, *χ2*(1) = 1.55, *p adj* = .382.

**Dislike Sexed Body (male).** The three-way interaction between Condition, Group, and dislike of one’s own sexed body (man) was negligible, *χ2*(2) = 0.23, *p adj* < .927, as well as the two-way interactions involving the covariate (all *ps* > .649). The two-way interaction between Condition and Group did not reach significance, *χ2*(2) = 0.98, *p adj* = .745, nor we found significant main effects of Condition, *χ2*(2) = 6.16, *p adj* = .096, and Group, *χ2*(1) = 0.75, *p adj* = .623. The effect of dislike of one’s own sexed body (man) was also not significant, *χ2*(1) = 0.26, *p adj* = .745.

**Wish to be the body of the "other" sex.** The three-way interaction between Condition, Group, and wishing to be the body of the "other" sex was negligible, *χ2*(2) = 1.17, *p adj* < .742, as well as the two-way interactions involving the covariate (all *ps* > .116). The two-way interaction between Condition and Group did not reach significance, *χ2*(2) = 1.61, *p adj* = .649. However, we found significant main effects of Condition, *χ2*(2) = 8.92, *p adj* = .025, and Group,*χ2*(1) = 7.31, *p adj* = .015. The covariate showed no significant main effect, *χ2*(1) = 0.28, *p adj* = .745.

**Pressure to be a "proper" woman.** The three-way interaction between Condition, Group, and pressure to be a "proper" woman was negligible, *χ2*(2) = 1.59, *p adj* < .649, as well as the two-way interactions involving the covariate (all *ps* > .578). The model yielded a significant two-way interaction between Condition and Group, *χ2*(2) = 39.95, *p adj* <.001, as well as significant main effects of Condition, *χ2*(2) = 54.37, *p adj* < .001, and Group, *χ2*(1) = 12.70, *p adj* = .001. The pressure to be a "proper" woman subscale showed no significant main effect, *χ2*(1) = 1.99, *p adj* = .302.

**Pressure to be a "proper" man.** The three-way interaction between Condition, Group, and pressure to be a "proper" man was negligible, *χ2*(2) = 3.46, *p adj* < .321, as well as the two-way interactions involving the covariate (all *ps* > .184). The model yielded a significant two-way interaction between Condition and Group, *χ2*(2) = 14.46, *p adj* = .002, as well as significant main effects of Condition, *χ2*(2) = 26.17, *p adj* < .001, and Group, *χ2*(1) = 11.03, *p adj* = .002. The pressure to be a "proper" man subscale showed no significant main effect, *χ2*(1) = 0.00, *p adj* = .995.

**Would be better to live as a man rather than as a woman.** The three-way interaction between Condition, Group, and better live (woman), *χ2*(2) = 2.04, *p adj* < .619, as well as the two-way interactions involving the covariate (all *ps* > .270). The model yielded a significant two-way interaction between Condition and Group, *χ2*(2) = 49.33, *p adj* < .001, as well as significant main effects of Condition, *χ2*(2) = 57.69, *p adj* < .001, and *χ2*(1) = 22.98, *p adj* < .001. The Better Live (woman) covariate showed no significant main effect, *χ2*(1) = 0.81, *p adj* = .619.

**Would be better to live as a woman rather than as a man.** The three-way interaction between Condition, Group, and better live (woman), *χ2*(2) = 1.80, *p adj* < .627, as well as the two-way interactions involving the covariate (all *ps* > .649). The model yielded a significant two-way interaction between Condition and Group, *χ2*(2) = 58.94, *p adj* <.001, as well as significant main effects of Condition, *χ2*(2) = 64.33, *p adj* < .001, and Group, *χ2*(1) = 26.51, *p adj* < .001. The Better Live (man) covariate showed no significant main effect, *χ2*(1) = 0.07, *p adj* = .871.

### **Gender Minority Stress and Resilience Measure (GMSR)**

**Victimization.** The two-way interaction between Condition and Victimization was not significant, *χ2*(1) = 1.27, *p adj* = .615. The model indicated a significant main effect of Condition, *χ2*(2) = 86.37, *p adj* <.001, while the main effect of Victimization was not statistically significant, *χ2*(1) = 1.66, *p adj* = .253.

**Rejection.** The two-way interaction between Condition and Rejection was not significant, χ2(1) = 0.52, *p adj* = .813. The model indicated a significant main effect of Condition, *χ2*(2) = 86.10, *p adj* <.001, while the main effect of Victimization was not statistically significant, *χ2*(1) = 0.18, *p adj* = .729.

**Nonaffirmation.** The two-way interaction between Condition and Nonaffirmation was not significant, *χ2*(1) = 2.01, *p adj* = .454. The model indicated a significant main effect of Condition, *χ2*(1) = 86.64, *p adj* = < .001, and a significant main effect of Non-Affirmation, *χ2*(1) = 8.98, *p adj* = .005.

**Negative Expectations.** The two-way interaction between Condition and Negative Expectations was not significant, *χ2*(1) = 3.63, *p adj* = .234. The model indicated a significant main effect of Condition, *χ2*(2) = 87.23, *p adj* <.001, and a significant main effect of Negative Expectations, *χ2*(1) = 0.02, *p adj* = .894.

**Tables**

**Table S2.** *Table reporting regression estimates, confidence intervals, and p-values of predictors for the main model on VCS.*

| ***Predictors*** | ***Estimates*** | ***CI*** | ***p*** |
| --- | --- | --- | --- |
| (Intercept) | 34.55 | 32.98 – 36.12 | <0.001 |
| Group1 | -4.32 | -5.89 – -2.76 | <0.001 |
| Condition1 | 2.32 | 1.64 – 3.00 | <0.001 |
| Condition2 | -0.59 | -1.27 – 0.09 | 0.091 |
| Text 1 | -0.50 | -1.18 – 0.18 | 0.150 |
| Text 2 | 0.23 | -0.45 – 0.91 | 0.505 |
| Group1 × Condition1 | 2.37 | 1.69 – 3.05 | <0.001 |
| Group1 × Condition2 | -0.95 | -1.63 – -0.27 | 0.006 |
| Group1 × Text 1 | 0.26 | -0.42 – 0.94 | 0.458 |
| Group1 × Text 2 | 0.34 | -0.34 – 1.03 | 0.321 |
| Condition1 × Text 1 | 0.42 | -0.54 – 1.39 | 0.387 |
| Condition2 × Text 1 | -0.21 | -1.18 – 0.75 | 0.665 |
| Condition1 × Text 2 | -0.17 | -1.13 – 0.79 | 0.728 |
| Condition2 × Text 2 | -0.35 | -1.32 – 0.61 | 0.473 |
| Group1 × Condition1 × Text 1 | 0.02 | -0.95 – 0.98 | 0.975 |
| Group1 × Condition2 × Text 1 | -0.03 | -0.99 – 0.93 | 0.951 |
| Group1 × Condition1 × Text 2 | -0.23 | -1.20 – 0.73 | 0.638 |
| Group1 × Condition2 × Text 2 | 0.22 | -0.74 – 1.19 | 0.649 |
| Random Effects | | | |
| σ^2^ | 23.79 | | |
| τ_00_ _Unique_Code_ | 25.27 | | |
| ICC | 0.52 | | |
| N _Unique_Code_ | 44 | | |
| Observations | 396 | | |
| Marginal R^2^ / Conditional R^2^ | 0.337 / 0.679 | | |

***Note****.* For this model and the subsequent model including questionnaire subscales as covariates, all categorical predictors were effects coded. Condition was coded as Silent Reading (1, 0), Reading Aloud (0, 1), and Listening (−1, −1). Group was coded as TGNC = 1 and Cisgender = −1. Text (where present) was coded as Neuter (1, 0), Feminine (0, 1), and Masculine (−1, −1). The intercept represents the grand mean.

**Table S3.** *Table reporting regression estimates, confidence intervals, and p-values of predictors for the model on VCS including Emotional Awareness as a covariate.*

| ***Predictors*** | ***Estimates*** | ***CI*** | ***p*** |
| --- | --- | --- | --- |
| (Intercept) | 34.41 | 32.82 – 36.01 | <0.001 |
| Group1 | -4.29 | -5.88 – -2.69 | <0.001 |
| Condition1 | 2.36 | 1.79 – 2.94 | <0.001 |
| Condition2 | -0.60 | -1.18 – -0.03 | 0.040 |
| Emotional Awareness | 0.48 | -0.83 – 1.80 | 0.470 |
| Group1 × Condition1 | 2.31 | 1.73 – 2.89 | <0.001 |
| Group1 × Condition2 | -0.94 | -1.51 – -0.36 | 0.002 |
| Group1 × Emotional Awareness | 0.21 | -1.11 – 1.52 | 0.757 |
| Condition1 × Emotional Awareness | 1.04 | 0.56 – 1.51 | <0.001 |
| Condition2 × Emotional Awareness | -0.39 | -0.87 – 0.08 | 0.103 |
| (Group1 × Condition1) × Emotional Awareness | 1.11 | 0.64 – 1.59 | <0.001 |
| (Group1 × Condition2) × Emotional Awareness | -0.63 | -1.11 – -0.16 | 0.009 |
| Random Effects | | | |
| σ^2^ | 22.76 | | |
| τ_00_ _Unique_Code_ | 27.08 | | |
| ICC | 0.54 | | |
| N _Unique_Code_ | 44 | | |
| Observations | 528 | | |
| Marginal R^2^ / Conditional R^2^ | 0.344 / 0.700 | | |

**Table S4.** *Table reporting regression estimates, confidence intervals, and p-values of predictors for the model on VCS including Noticing as a covariate.*

| ***Predictors*** | ***Estimates*** | ***CI*** | ***p*** |
| --- | --- | --- | --- |
| (Intercept) | 34.28 | 32.71 – 35.85 | <0.001 |
| Group1 | -4.32 | -5.89 – -2.75 | <0.001 |
| Condition1 | 2.46 | 1.85 – 3.06 | <0.001 |
| Condition2 | -0.67 | -1.27 – -0.07 | 0.030 |
| Noticing | -0.44 | -2.13 – 1.24 | 0.607 |
| Group1 × Condition1 | 2.43 | 1.83 – 3.04 | <0.001 |
| Group1 × Condition2 | -0.96 | -1.56 – -0.36 | 0.002 |
| Group1 × Noticing | -1.04 | -2.72 – 0.65 | 0.227 |
| Condition1 ×Noticing | 0.46 | -0.18 – 1.11 | 0.158 |
| Condition2 ×Noticing | -0.02 | -0.66 – 0.63 | 0.954 |
| (Group1 × Condition1) × Noticing | 0.21 | -0.43 – 0.86 | 0.516 |
| (Group1 × Condition2) × Noticing | -0.21 | -0.85 – 0.44 | 0.525 |
| Random Effects | | | |
| σ2 | 24.28 | | |
| τ00 Unique_Code | 25.55 | | |
| ICC | 0.51 | | |
| N Unique_Code | 44 | | |
| Observations | 528 | | |
| Marginal R2 / Conditional R2 | 0.343 / 0.680 | | |

**Table S5.** *Table reporting regression estimates, confidence intervals, and p-values of predictors for the model on VCS including Attention Regulation as a covariate.*

| **Predictors** | **Estimates** | **CI** | **p** |
| --- | --- | --- | --- |
| (Intercept) | 34.49 | 32.91 – 36.07 | <0.001 |
| Group1 | -3.98 | -5.56 – -2.40 | <0.001 |
| Condition1 | 2.29 | 1.69 – 2.89 | <0.001 |
| Condition2 | -0.58 | -1.19 – 0.02 | 0.058 |
| Attention Regulation | 1.17 | -0.18 – 2.52 | 0.089 |
| Group1 × Condition1 | 2.21 | 1.61 – 2.82 | <0.001 |
| Group1 × Condition2 | -0.85 | -1.45 – -0.25 | 0.006 |
| Group1 × Attention Regulation | 0.29 | -1.06 – 1.63 | 0.676 |
| Condition1 × Attention Regulation | -0.66 | -1.18 – -0.15 | 0.012 |
| Condition2 × Attention Regulation | 0.45 | -0.06 – 0.97 | 0.087 |
| (Group1 × Condition1) × Attention Regulation | -0.57 | -1.09 – -0.06 | 0.029 |
| (Group1 × Condition2) × Attention Regulation | 0.24 | -0.28 – 0.75 | 0.362 |
| Random Effects | | | |
| σ2 | 23.88 | | |
| τ00 Unique_Code | 25.23 | | |
| ICC | 0.51 | | |
| N Unique_Code | 44 | | |
| Observations | 528 | | |
| Marginal R2 / Conditional R2 | 0.353 / 0.685 | | |

**Table S6.** *Table reporting regression estimates, confidence intervals, and p-values of predictors for the model on VCS including Not Distracting as a covariate.*

| **Predictors** | **Estimates** | **CI** | **p** |
| --- | --- | --- | --- |
| (Intercept) | 34.33 | 32.80 – 35.86 | <0.001 |
| Group1 | -4.51 | -6.04 – -2.98 | <0.001 |
| Condition1 | 2.50 | 1.90 – 3.11 | <0.001 |
| Condition2 | -0.68 | -1.28 – -0.08 | 0.028 |
| Not Distracting | -1.72 | -3.33 – -0.12 | 0.035 |
| Group1 × Condition1 | 2.42 | 1.81 – 3.02 | <0.001 |
| Group1 × Condition2 | -0.95 | -1.56 – -0.35 | 0.002 |
| Group1 × Not Distracting | -0.64 | -2.24 – 0.96 | 0.432 |
| Condition1 ×Not Distracting | 0.30 | -0.33 – 0.93 | 0.350 |
| Condition2 ×Not Distracting | 0.04 | -0.59 – 0.67 | 0.904 |
| (Group1 × Condition1) × Not Distracting | 0.52 | -0.11 – 1.15 | 0.108 |
| (Group1 × Condition2) × Not Distracting | -0.26 | -0.89 – 0.37 | 0.421 |
| Random Effects | | | |
| σ2 | 24.29 | | |
| τ00 Unique_Code | 23.99 | | |
| ICC | 0.50 | | |
| N Unique_Code | 44 | | |
| Observations | 528 | | |
| Marginal R2 / Conditional R2 | 0.362 / 0.679 | | |

**Table S7.** *Table reporting regression estimates, confidence intervals, and p-values of predictors for the model on VCS including Not Worrying as a covariate.*

| **Predictors** | **Estimates** | **CI** | **p** |
| --- | --- | --- | --- |
| (Intercept) | 34.66 | 33.19 – 36.14 | <0.001 |
| Group1 | -4.08 | -5.55 – -2.60 | <0.001 |
| Condition1 | 2.30 | 1.70 – 2.89 | <0.001 |
| Condition2 | -0.60 | -1.19 – 0.00 | 0.050 |
| Not Worrying | 1.14 | -0.27 – 2.56 | 0.113 |
| Group1 × Condition1 | 2.29 | 1.69 – 2.88 | <0.001 |
| Group1 × Condition2 | -0.94 | -1.53 – -0.34 | 0.002 |
| Group1 × Not Worrying | 1.50 | 0.08 – 2.91 | 0.038 |
| Condition1 ×Not Worrying | -0.52 | -1.10 – 0.05 | 0.073 |
| Condition2 × Not Worrying | 0.13 | -0.44 – 0.70 | 0.654 |
| (Group1 × Condition1) × Not Worrying | -0.83 | -1.40 – -0.26 | 0.005 |
| (Group1 × Condition2) × Not Worrying | 0.28 | -0.29 – 0.86 | 0.332 |
| Random Effects | | | |
| σ2 | 23.75 | | |
| τ00 Unique_Code | 22.17 | | |
| ICC | 0.48 | | |
| N Unique_Code | 44 | | |
| Observations | 528 | | |
| Marginal R2 / Conditional R2 | 0.392 / 0.686 | | |

**Table S8.** *Table reporting regression estimates, confidence intervals, and p-values of predictors for the model on VCS including Self Regulation as a covariate.*

| **Predictors** | **Estimates** | **CI** | **p** |
| --- | --- | --- | --- |
| (Intercept) | 34.85 | 33.21 – 36.48 | <0.001 |
| Group1 | -3.38 | -5.02 – -1.75 | <0.001 |
| Condition1 | 2.52 | 1.85 – 3.19 | <0.001 |
| Condition2 | -0.95 | -1.62 – -0.28 | 0.005 |
| Self Regulation | 1.67 | 0.24 – 3.11 | 0.022 |
| Group1 × Condition1 | 2.27 | 1.60 – 2.94 | <0.001 |
| Group1 × Condition2 | -0.98 | -1.65 – -0.31 | 0.004 |
| Group1 × Self Regulation | 0.81 | -0.63 – 2.24 | 0.269 |
| Condition1 × Self Regulation | -0.20 | -0.79 – 0.39 | 0.507 |
| Condition2 × Self Regulation | -0.04 | -0.63 – 0.54 | 0.882 |
| (Group1 × Condition1) × Self Regulation | 0.17 | -0.42 – 0.76 | 0.574 |
| (Group1 × Condition2) × Self Regulation | -0.60 | -1.18 – -0.01 | 0.047 |
| Random Effects | | | |
| σ2 | 24.19 | | |
| τ00 Unique_Code | 22.00 | | |
| ICC | 0.48 | | |
| N Unique_Code | 44 | | |
| Observations | 528 | | |
| Marginal R2 / Conditional R2 | 0.389 / 0.680 | | |

**Table S9.** *Table reporting regression estimates, confidence intervals, and p-values of predictors for the model on VCS including Body Listening as a covariate.*

| **Predictors** | **Estimates** | **CI** | **p** |
| --- | --- | --- | --- |
| (Intercept) | 34.67 | 33.03 – 36.30 | <0.001 |
| Group1 | -4.09 | -5.73 – -2.46 | <0.001 |
| Condition1 | 2.36 | 1.74 – 2.99 | <0.001 |
| Condition2 | -0.68 | -1.30 – -0.06 | 0.032 |
| Body Listening | 0.48 | -0.86 – 1.83 | 0.480 |
| Group1 × Condition1 | 2.42 | 1.80 – 3.04 | <0.001 |
| Group1 × Condition2 | -0.95 | -1.57 – -0.32 | 0.003 |
| Group1 × Body Listening | 0.71 | -0.63 – 2.06 | 0.299 |
| Condition1 × Body Listening | 0.15 | -0.36 – 0.66 | 0.562 |
| Condition2 × Body Listening | 0.04 | -0.47 – 0.55 | 0.882 |
| (Group1 × Condition1) × Body Listening | -0.19 | -0.70 – 0.32 | 0.474 |
| (Group1 × Condition2) × Body Listening | -0.12 | -0.63 – 0.39 | 0.646 |
| Random Effects | | | |
| σ2 | 24.40 | | |
| τ00 Unique_Code | 26.12 | | |
| ICC | 0.52 | | |
| N Unique_Code | 44 | | |
| Observations | 528 | | |
| Marginal R2 / Conditional R2 | 0.335 / 0.679 | | |

**Table S10.** *Table reporting regression estimates, confidence intervals, and p-values of predictors for the model on VCS including Trusting as a covariate.*

| **Predictors** | **Estimates** | **CI** | **p** |
| --- | --- | --- | --- |
| (Intercept) | 35.22 | 33.05 – 37.39 | <0.001 |
| Group1 | -3.45 | -5.62 – -1.28 | 0.002 |
| Condition1 | 1.18 | 0.40 – 1.96 | 0.003 |
| Condition2 | -0.06 | -0.84 – 0.72 | 0.881 |
| Trusting | 0.86 | -0.76 – 2.48 | 0.300 |
| Group1 × Condition1 | 0.85 | 0.07 – 1.63 | 0.034 |
| Group1 × Condition2 | -0.17 | -0.95 – 0.61 | 0.665 |
| Group1 × Trusting | 0.84 | -0.78 – 2.46 | 0.307 |
| Condition1 × Trusting | -1.62 | -2.20 – -1.03 | <0.001 |
| Condition2 × Trusting | 0.83 | 0.25 – 1.42 | 0.005 |
| (Group1 × Condition1) × Trusting | -1.32 | -1.91 – -0.74 | <0.001 |
| (Group1 × Condition2) × Trusting | 0.62 | 0.03 – 1.20 | 0.039 |
| Random Effects | | | |
| σ2 | 21.08 | | |
| τ00 Unique_Code | 25.38 | | |
| ICC | 0.55 | | |
| N Unique_Code | 44 | | |
| Observations | 528 | | |
| Marginal R2 / Conditional R2 | 0.387 / 0.722 | | |

**Table S11.** *Table reporting regression estimates, confidence intervals, and p-values of predictors for the model on VCS including Cognitive Reappraisal as a covariate.*

| **Predictors** | **Estimates** | **CI** | **p** |
| --- | --- | --- | --- |
| (Intercept) | 34.38 | 32.83 – 35.94 | <0.001 |
| Group1 | -4.04 | -5.60 – -2.49 | <0.001 |
| Condition1 | 2.51 | 1.91 – 3.11 | <0.001 |
| Condition2 | -0.70 | -1.30 – -0.10 | 0.023 |
| Cognitive Reappraisal | 1.34 | -0.09 – 2.77 | 0.066 |
| Group1 × Condition1 | 2.43 | 1.82 – 3.03 | <0.001 |
| Group1 × Condition2 | -1.01 | -1.61 – -0.41 | 0.001 |
| Group1 × Cognitive Reappraisal | -0.25 | -1.68 – 1.18 | 0.732 |
| Condition1 × Cognitive Reappraisal | 0.34 | -0.21 – 0.90 | 0.222 |
| Condition2 × Cognitive Reappraisal | -0.32 | -0.87 – 0.23 | 0.250 |
| (Group1 × Condition1) × Cognitive Reappraisal | 0.50 | -0.05 – 1.06 | 0.073 |
| (Group1 × Condition2) × Cognitive Reappraisal | -0.36 | -0.92 – 0.19 | 0.194 |
| Random Effects | | | |
| σ2 | 24.21 | | |
| τ00 Unique_Code | 25.05 | | |
| ICC | 0.51 | | |
| N Unique_Code | 44 | | |
| Observations | 528 | | |
| Marginal R2 / Conditional R2 | 0.350 / 0.681 | | |

**Table S12.** *Table reporting regression estimates, confidence intervals, and p-values of predictors for the model on VCS including Expressive Suppression as a covariate.*

| **Predictors** | **Estimates** | **CI** | **p** |
| --- | --- | --- | --- |
| (Intercept) | 34.32 | 32.69 – 35.95 | <0.001 |
| Group1 | -4.43 | -6.06 – -2.80 | <0.001 |
| Condition1 | 2.43 | 1.82 – 3.05 | <0.001 |
| Condition2 | -0.67 | -1.29 – -0.06 | 0.032 |
| Expressive Suppression | 0.68 | -0.78 – 2.15 | 0.361 |
| Group1 × Condition1 | 2.30 | 1.68 – 2.91 | <0.001 |
| Group1 × Condition2 | -0.88 | -1.49 – -0.26 | 0.005 |
| Group1 × Expressive Suppression | 0.42 | -1.05 – 1.89 | 0.573 |
| Condition1 × Expressive Suppression | 0.29 | -0.26 – 0.84 | 0.306 |
| Condition2 × Expressive Suppression | -0.31 | -0.87 – 0.24 | 0.263 |
| (Group1 × Condition1) × Expressive Suppression | -0.02 | -0.57 – 0.53 | 0.935 |
| (Group1 × Condition2) × Expressive Suppression | 0.13 | -0.43 – 0.68 | 0.653 |
| Random Effects | | | |
| σ2 | 24.39 | | |
| τ00 Unique_Code | 26.65 | | |
| ICC | 0.52 | | |
| N Unique_Code | 44 | | |
| Observations | 528 | | |
| Marginal R2 / Conditional R2 | 0.328 / 0.679 | | |

**Table S13.** *Table reporting regression estimates, confidence intervals, and p-values of predictors for the model on VCS including Difficulty Identifying Feeling* *as a covariate.*

| **Predictors** | **Estimates** | **CI** | **p** |
| --- | --- | --- | --- |
| (Intercept) | 35.30 | 33.45 – 37.14 | <0.001 |
| Group1 | -3.59 | -5.43 – -1.75 | <0.001 |
| Condition1 | 2.29 | 1.57 – 3.01 | <0.001 |
| Condition2 | -0.64 | -1.36 – 0.08 | 0.082 |
| DiF | -0.18 | -0.46 – 0.10 | 0.208 |
| Group1 × Condition1 | 1.75 | 1.03 – 2.47 | <0.001 |
| Group1 × Condition2 | -0.55 | -1.26 – 0.17 | 0.136 |
| Group1 × DiF | -0.23 | -0.51 – 0.05 | 0.101 |
| Condition1 × DiF | 0.17 | 0.06 – 0.28 | 0.003 |
| Condition2 × DiF | -0.11 | -0.22 – -0.00 | 0.047 |
| (Group1 × Condition1) × DiF | 0.04 | -0.07 – 0.15 | 0.506 |
| (Group1 × Condition2) × DiF | -0.00 | -0.11 – 0.11 | 0.988 |
| Random Effects | | | |
| σ2 | 23.98 | | |
| τ00 Unique_Code | 24.32 | | |
| ICC | 0.50 | | |
| N Unique_Code | 44 | | |
| Observations | 528 | | |
| Marginal R2 / Conditional R2 | 0.362 / 0.683 | | |

**Table S14.** *Table reporting regression estimates, confidence intervals, and p-values of predictors for the model on VCS including Difficulty Describing Feelings* *as a covariate.*

| **Predictors** | **Estimates** | **CI** | **p** |
| --- | --- | --- | --- |
| (Intercept) | 35.14 | 33.60 – 36.68 | <0.001 |
| Group1 | -4.02 | -5.57 – -2.48 | <0.001 |
| Condition1 | 2.52 | 1.89 – 3.16 | <0.001 |
| Condition2 | -0.69 | -1.33 – -0.05 | 0.035 |
| DDF | -0.14 | -0.48 – 0.19 | 0.410 |
| Group1 × Condition1 | 2.10 | 1.46 – 2.74 | <0.001 |
| Group1 × Condition2 | -0.82 | -1.46 – -0.18 | 0.012 |
| Group1 × DDF | -0.43 | -0.77 – -0.09 | 0.012 |
| Condition1 × DDF | 0.16 | 0.02 – 0.30 | 0.022 |
| Condition2 × DDF | -0.09 | -0.22 – 0.05 | 0.229 |
| (Group1 × Condition1) × DDF | -0.06 | -0.20 – 0.08 | 0.416 |
| (Group1 × Condition2) × DDF | 0.03 | -0.11 – 0.17 | 0.685 |
| Random Effects | | | |
| σ2 | 24.23 | | |
| τ00 Unique_Code | 21.59 | | |
| ICC | 0.47 | | |
| N Unique_Code | 44 | | |
| Observations | 528 | | |
| Marginal R2 / Conditional R2 | 0.393 / 0.679 | | |

**Table S15.** *Table reporting regression estimates, confidence intervals, and p-values of predictors for the model on VCS including Externally-Oriented Thinking* *as a covariate.*

| **Predictors** | **Estimates** | **CI** | **p** |
| --- | --- | --- | --- |
| (Intercept) | 34.50 | 32.93 – 36.06 | <0.001 |
| Group1 | -4.26 | -5.82 – -2.69 | <0.001 |
| Condition1 | 2.48 | 1.89 – 3.07 | <0.001 |
| Condition2 | -0.66 | -1.26 – -0.07 | 0.028 |
| EOT | 0.01 | -0.41 – 0.42 | 0.970 |
| Group1 × Condition1 | 2.40 | 1.80 – 2.99 | <0.001 |
| Group1 × Condition2 | -0.97 | -1.56 – -0.38 | 0.001 |
| Group1 × EOT | -0.32 | -0.74 – 0.10 | 0.132 |
| Condition1 × EOT | -0.10 | -0.26 – 0.05 | 0.193 |
| Condition2 × EOT | 0.05 | -0.11 – 0.21 | 0.545 |
| (Group1 × Condition1) × EOT | -0.22 | -0.38 – -0.07 | 0.006 |
| (Group1 × Condition2) × EOT | 0.10 | -0.05 – 0.26 | 0.198 |
| Random Effects | | | |
| σ2 | 23.97 | | |
| τ00 Unique_Code | 25.80 | | |
| ICC | 0.52 | | |
| N Unique_Code | 44 | | |
| Observations | 528 | | |
| Marginal R2 / Conditional R2 | 0.344 / 0.684 | | |

**Table S16.** *Table reporting regression estimates, confidence intervals, and p-values of predictors for the model on VCS including Total TAS-20*  *as a covariate.*

| **Predictors** | **Estimates** | **CI** | **p** |
| --- | --- | --- | --- |
| (Intercept) | 35.42 | 33.79 – 37.04 | <0.001 |
| Group1 | -3.73 | -5.35 – -2.11 | <0.001 |
| Condition1 | 2.53 | 1.86 – 3.20 | <0.001 |
| Condition2 | -0.71 | -1.38 – -0.04 | 0.038 |
| Total Tas-20 | -0.09 | -0.22 – 0.04 | 0.161 |
| Group1 × Condition1 | 2.08 | 1.41 – 2.75 | <0.001 |
| Group1 × Condition2 | -0.77 | -1.44 – -0.10 | 0.024 |
| Group1 × Total Tas-20 | -0.18 | -0.31 – -0.05 | 0.008 |
| Condition1 × Total Tas-20 | 0.05 | -0.00 – 0.11 | 0.061 |
| Condition2 × Total Tas-20 | -0.03 | -0.09 – 0.02 | 0.224 |
| (Group1 × Condition1) × Total Tas-20 | -0.02 | -0.07 – 0.04 | 0.519 |
| (Group1 × Condition2) × tTotal Tas-20 | 0.01 | -0.04 – 0.07 | 0.668 |
| Random Effects | | | |
| σ2 | 24.30 | | |
| τ00 Unique_Code | 21.77 | | |
| ICC | 0.47 | | |
| N Unique_Code | 44 | | |
| Observations | 528 | | |
| Marginal R2 / Conditional R2 | 0.390 / 0.678 | | |

**Table S17.** *Table reporting regression estimates, confidence intervals, and p-values of predictors for the model on VCS including Satisfaction being the affirmed gender (women)* *as a covariate.*

| **Predictors** | **Estimates** | **CI** | **p** |
| --- | --- | --- | --- |
| (Intercept) | 33.88 | 31.94 – 35.83 | <0.001 |
| Group1 | -3.68 | -5.63 – -1.73 | <0.001 |
| Condition1 | 2.86 | 2.10 – 3.62 | <0.001 |
| Condition2 | -1.03 | -1.79 – -0.27 | 0.008 |
| Satisfaction Affirmed Gender (women) | -0.55 | -1.78 – 0.68 | 0.382 |
| Group1 × Condition1 | 2.59 | 1.83 – 3.35 | <0.001 |
| Group1 × Condition2 | -0.97 | -1.73 – -0.21 | 0.013 |
| Group1 × Satisfaction Affirmed Gender (women) | -0.08 | -1.31 – 1.15 | 0.896 |
| Condition1 × Satisfaction Affirmed Gender (women) | -0.19 | -0.67 – 0.29 | 0.440 |
| Condition2 × Satisfaction Affirmed Gender (women) | -0.11 | -0.59 – 0.37 | 0.653 |
| (Group1 × Condition1) × Satisfaction Affirmed Gender (women) | 0.04 | -0.44 – 0.52 | 0.884 |
| (Group1 × Condition2) × Satisfaction Affirmed Gender (women) | 0.05 | -0.43 – 0.53 | 0.846 |
| Random Effects | | | |
| σ2 | 24.74 | | |
| τ00 Unique_Code | 24.98 | | |
| ICC | 0.50 | | |
| N Unique_Code | 31 | | |
| Observations | 372 | | |
| Marginal R2 / Conditional R2 | 0.331 / 0.667 | | |

**Table S18.** *Table reporting regression estimates, confidence intervals, and p-values of predictors for the model on VCS including Satisfaction being the affirmed gender (men)* *as a covariate.*

| **Predictors** | **Estimates** | **CI** | **p** |
| --- | --- | --- | --- |
| (Intercept) | 34.80 | 32.67 – 36.94 | <0.001 |
| Group1 | -5.38 | -7.51 – -3.25 | <0.001 |
| Condition1 | 2.53 | 1.59 – 3.47 | <0.001 |
| Condition2 | -0.64 | -1.58 – 0.30 | 0.179 |
| Satisfaction Affirmed Gender (men) | 0.56 | -0.81 – 1.93 | 0.424 |
| Group1 × Condition1 | 2.30 | 1.36 – 3.24 | <0.001 |
| Group1 × Condition2 | -0.99 | -1.93 – -0.05 | 0.039 |
| Group1 × Satisfaction Affirmed Gender (men) | 0.37 | -1.00 – 1.74 | 0.598 |
| Condition1 × Satisfaction Affirmed Gender (men) | -0.15 | -0.75 – 0.45 | 0.630 |
| Condition2 × Satisfaction Affirmed Gender (men) | 0.03 | -0.57 – 0.63 | 0.921 |
| (Group1 × Condition1) × Satisfaction Affirmed Gender (men) | -0.08 | -0.68 – 0.52 | 0.787 |
| (Group1 × Condition2) × Satisfaction Affirmed Gender (men) | 0.12 | -0.48 – 0.72 | 0.688 |
| Random Effects | | | |
| σ2 | 29.92 | | |
| τ00 Unique_Code | 23.29 | | |
| ICC | 0.44 | | |
| N Unique_Code | 29 | | |
| Observations | 348 | | |
| Marginal R2 / Conditional R2 | 0.385 / 0.654 | | |

**Table S19.** *Table reporting regression estimates, confidence intervals, and p-values of predictors for the model on VCS including Wishing to be the “other” gender (women)* *as a covariate.*

| **Predictors** | **Estimates** | **CI** | **p** |
| --- | --- | --- | --- |
| (Intercept) | 34.66 | 31.90 – 37.41 | <0.001 |
| Group1 | -4.10 | -6.86 – -1.35 | 0.004 |
| Condition1 | 2.36 | 1.41 – 3.31 | <0.001 |
| Condition2 | -0.63 | -1.58 – 0.31 | 0.190 |
| Wish Other Gender (women) | 0.69 | -1.30 – 2.68 | 0.494 |
| Group1 × Condition1 | 2.70 | 1.76 – 3.65 | <0.001 |
| Group1 × Condition2 | -1.24 | -2.19 – -0.29 | 0.011 |
| Group1 × Wish Other Gender (women) | -0.87 | -2.86 – 1.12 | 0.390 |
| Condition1 ×Wish Other Gender (women) | 0.23 | -0.45 – 0.92 | 0.503 |
| Condition2 × Wish Other Gender (women) | -0.14 | -0.82 – 0.54 | 0.686 |
| (Group1 × Condition1) × Wish Other Gender (women) | -0.31 | -0.99 – 0.37 | 0.375 |
| (Group1 × Condition2) × Wish Other Gender (women) | 0.03 | -0.65 – 0.71 | 0.936 |
| Random Effects | | | |
| σ2 | 24.00 | | |
| τ00 Unique_Code | 31.84 | | |
| ICC | 0.57 | | |
| N Unique_Code | 28 | | |
| Observations | 336 | | |
| Marginal R2 / Conditional R2 | 0.345 / 0.718 | | |

**Table S20.** *Table reporting regression estimates, confidence intervals, and p-values of predictors for the model on VCS including Wishing to be the “other” gender (men)* *as a covariate.*

| **Predictors** | **Estimates** | **CI** | **p** |
| --- | --- | --- | --- |
| (Intercept) | 35.52 | 33.53 – 37.50 | <0.001 |
| Group1 | -4.72 | -6.70 – -2.73 | <0.001 |
| Condition1 | 2.64 | 1.86 – 3.43 | <0.001 |
| Condition2 | -0.71 | -1.50 – 0.08 | 0.077 |
| Wish Other Gender (men) | -0.41 | -1.60 – 0.79 | 0.505 |
| Group1 × Condition1 | 2.04 | 1.25 – 2.83 | <0.001 |
| Group1 × Condition2 | -0.95 | -1.74 – -0.16 | 0.018 |
| Group1 × Wish Other Gender (men) | 1.49 | 0.30 – 2.69 | 0.015 |
| Condition1 ×Wish Other Gender (men) | 0.09 | -0.38 – 0.56 | 0.704 |
| Condition2 × Wish Other Gender (men) | -0.25 | -0.72 – 0.22 | 0.302 |
| (Group1 × Condition1) × Wish Other Gender (men) | 0.08 | -0.39 – 0.55 | 0.740 |
| (Group1 × Condition2) × Wish Other Gender (men) | 0.11 | -0.36 – 0.59 | 0.635 |
| Random Effects | | | |
| σ2 | 28.29 | | |
| τ00 Unique_Code | 27.67 | | |
| ICC | 0.49 | | |
| N Unique_Code | 33 | | |
| Observations | 396 | | |
| Marginal R2 / Conditional R2 | 0.360 / 0.676 | | |

**Table S21.** *Table reporting regression estimates, confidence intervals, and p-values of predictors for the model on VCS including* Dislike of one's own sexed body (female-assigned) *as a covariate.*

| **Predictors** | **Estimates** | **CI** | **p** |
| --- | --- | --- | --- |
| (Intercept) | 35.20 | 32.89 – 37.52 | <0.001 |
| Group1 | -3.44 | -5.75 – -1.12 | 0.004 |
| Condition1 | 1.92 | 0.92 – 2.93 | <0.001 |
| Condition2 | -0.57 | -1.58 – 0.43 | 0.262 |
| Dislike Sexed Body (female) | 0.82 | -0.48 – 2.12 | 0.214 |
| Group1 × Condition1 | 2.26 | 1.25 – 3.26 | <0.001 |
| Group1 × Condition2 | -1.06 | -2.06 – -0.05 | 0.039 |
| Group1 × Dislike Sexed Body (female) | 0.91 | -0.39 – 2.21 | 0.170 |
| Condition1 × Dislike Sexed Body (female) | -0.11 | -0.67 – 0.45 | 0.699 |
| Condition2 × Dislike Sexed Body (female) | -0.08 | -0.64 – 0.48 | 0.784 |
| (Group1 × Condition1) × Dislike Sexed Body (female) | -0.26 | -0.82 – 0.31 | 0.370 |
| (Group1 × Condition2) × Dislike Sexed Body (female) | 0.04 | -0.52 – 0.61 | 0.875 |
| Random Effects | | | |
| σ2 | 30.36 | | |
| τ00 Unique_Code | 24.35 | | |
| ICC | 0.45 | | |
| N Unique_Code | 30 | | |
| Observations | 360 | | |
| Marginal R2 / Conditional R2 | 0.339 / 0.633 | | |

**Table S22.** *Table reporting regression estimates, confidence intervals, and p-values of predictors for the model on VCS including* Dislike of one's own sexed body (male-assigned) *as a covariate.*

| **Predictors** | **Estimates** | **CI** | **p** |
| --- | --- | --- | --- |
| (Intercept) | 33.59 | 26.82 – 40.37 | <0.001 |
| Group1 | -2.98 | -9.76 – 3.79 | 0.387 |
| Condition1 | 2.64 | 0.49 – 4.79 | 0.016 |
| Condition2 | -0.77 | -2.92 – 1.38 | 0.479 |
| Dislike Sexed Body (male) | 1.68 | -4.79 – 8.15 | 0.610 |
| Group1 × Condition1 | 1.08 | -1.07 – 3.23 | 0.325 |
| Group1 × Condition2 | -0.63 | -2.78 – 1.52 | 0.566 |
| Group1 × Dislike Sexed Body (male) | -1.52 | -7.99 – 4.95 | 0.645 |
| Condition1 × Dislike Sexed Body (male) | -1.31 | -3.36 – 0.74 | 0.210 |
| Condition2 × Dislike Sexed Body (male) | 0.47 | -1.58 – 2.52 | 0.654 |
| (Group1 × Condition1) × Dislike Sexed Body (male) | 0.50 | -1.55 – 2.55 | 0.634 |
| (Group1 × Condition2) × Dislike Sexed Body (male) | -0.31 | -2.36 – 1.74 | 0.767 |
| Random Effects | | | |
| σ2 | 23.03 | | |
| τ00 Unique_Code | 36.25 | | |
| ICC | 0.61 | | |
| N Unique_Code | 25 | | |
| Observations | 300 | | |
| Marginal R2 / Conditional R2 | 0.315 / 0.734 | | |

**Table S23.** *Table reporting regression estimates, confidence intervals, and p-values of predictors for the model on VCS including Wishing to have the body of the “other” sex* *as a covariate.*

| **Predictors** | **Estimates** | **CI** | **p** |
| --- | --- | --- | --- |
| (Intercept) | 35.88 | 32.12 – 39.65 | <0.001 |
| Group1 | -5.19 | -8.95 – -1.42 | 0.007 |
| Condition1 | 1.83 | 0.46 – 3.19 | 0.009 |
| Condition2 | -0.07 | -1.43 – 1.30 | 0.924 |
| Wish Other Sexed Body | -0.76 | -3.54 – 2.03 | 0.594 |
| Group1 × Condition1 | 0.88 | -0.48 – 2.24 | 0.205 |
| Group1 × Condition2 | -0.38 | -1.74 – 0.98 | 0.582 |
| Group1 × Wish Other Sexed Body | 1.19 | -1.59 – 3.97 | 0.402 |
| Condition1 × Wish Other Sexed Body | -1.22 | -2.22 – -0.21 | 0.018 |
| Condition2 × Wish Other Sexed Body | 0.47 | -0.54 – 1.48 | 0.359 |
| (Group1 × Condition1) × other sexed body | -0.49 | -1.50 – 0.51 | 0.338 |
| (Group1 × Condition2) × Wish Other Sexed Body | 0.47 | -0.54 – 1.47 | 0.361 |
| Random Effects | | | |
| σ2 | 22.59 | | |
| τ00 Unique_Code | 26.91 | | |
| ICC | 0.54 | | |
| N Unique_Code | 44 | | |
| Observations | 528 | | |
| Marginal R2 / Conditional R2 | 0.348 / 0.703 | | |

**Table S24.** *Table reporting regression estimates, confidence intervals, and p-values of predictors for the model on VCS including Pressure to be a “proper” woman* *as a covariate.*

| **Predictors** | **Estimates** | **CI** | **p** |
| --- | --- | --- | --- |
| (Intercept) | 33.80 | 31.84 – 35.75 | <0.001 |
| Group1 | -3.55 | -5.51 – -1.59 | <0.001 |
| Condition1 | 2.49 | 1.81 – 3.18 | <0.001 |
| Condition2 | -0.75 | -1.43 – -0.06 | 0.032 |
| Pressure (women) | 1.11 | -0.43 – 2.65 | 0.159 |
| Group1 × Condition1 | 2.17 | 1.49 – 2.85 | <0.001 |
| Group1 × Condition2 | -0.82 | -1.50 – -0.14 | 0.019 |
| Group1 × Pressure (women) | -0.77 | -2.31 – 0.78 | 0.329 |
| Condition1 × Pressure (woman) | -0.08 | -0.62 – 0.45 | 0.761 |
| Condition2 × Pressure (woman) | -0.10 | -0.64 – 0.44 | 0.718 |
| (Group1 × Condition1) × Pressure (woman) | -0.34 | -0.88 – 0.20 | 0.216 |
| (Group1 × Condition2) × Pressure (woman) | 0.11 | -0.42 – 0.65 | 0.677 |
| Random Effects | | | |
| σ2 | 22.09 | | |
| τ00 Unique_Code | 28.47 | | |
| ICC | 0.56 | | |
| N Unique_Code | 33 | | |
| Observations | 396 | | |
| Marginal R2 / Conditional R2 | 0.315 / 0.701 | | |

**Table S25.** *Table reporting regression estimates, confidence intervals, and p-values of predictors for the model on VCS including Pressure to be a “proper” man* *as a covariate.*

| **Predictors** | **Estimates** | **CI** | **p** |
| --- | --- | --- | --- |
| (Intercept) | 36.10 | 33.38 – 38.82 | <0.001 |
| Group1 | -4.59 | -7.31 – -1.87 | 0.001 |
| Condition1 | 2.62 | 1.58 – 3.66 | <0.001 |
| Condition2 | -0.70 | -1.74 – 0.34 | 0.188 |
| Pressure (man) | 0.01 | -2.46 – 2.48 | 0.995 |
| Group1 × Condition1 | 2.01 | 0.97 – 3.05 | <0.001 |
| Group1 × Condition2 | -1.05 | -2.10 – -0.01 | 0.047 |
| Group1 × Pressure (man) | 2.12 | -0.35 – 4.58 | 0.093 |
| Condition1 × Pressure (man) | -0.08 | -1.03 – 0.87 | 0.868 |
| Condition2 × Pressure (man) | -0.38 | -1.32 – 0.57 | 0.435 |
| (Group1 × Condition1) × Pressure (man) | 0.87 | -0.07 – 1.82 | 0.070 |
| (Group1 × Condition2) × Pressure (man) | -0.26 | -1.21 – 0.68 | 0.584 |
| Random Effects | | | |
| σ2 | 25.20 | | |
| τ00 Unique_Code | 26.55 | | |
| ICC | 0.51 | | |
| N Unique_Code | 32 | | |
| Observations | 384 | | |
| Marginal R2 / Conditional R2 | 0.364 / 0.690 | | |

**Table S26.** *Table reporting regression estimates, confidence intervals, and p-values of predictors for the model on VCS including Better Live as a woman than as man as* *a covariate.*

| Predictors | Estimates | CI | p |
| --- | --- | --- | --- |
| (Intercept) | 34.36 | 32.71 – 36.02 | <0.001 |
| Group1 | -4.04 | -5.70 – -2.39 | <0.001 |
| Condition1 | 2.33 | 1.71 – 2.94 | <0.001 |
| Condition2 | -0.65 | -1.27 – -0.03 | 0.039 |
| Better Live (woman) | 0.52 | -0.62 – 1.67 | 0.366 |
| Group1 × Condition1 | 2.20 | 1.58 – 2.81 | <0.001 |
| Group1 × Condition2 | -0.86 | -1.48 – -0.24 | 0.007 |
| Group1 × Better Live (woman) | -0.15 | -1.29 – 0.99 | 0.793 |
| Condition1 × Better Live (woman) | -0.43 | -0.86 – -0.00 | 0.048 |
| Condition2 × Better Live (woman) | 0.25 | -0.18 – 0.67 | 0.257 |
| (Group1 × Condition1) × Better Live (woman) | -0.25 | -0.68 – 0.18 | 0.248 |
| (Group1 × Condition2) ×Better Live (woman) | -0.03 | -0.46 – 0.39 | 0.880 |
| Random Effects | | | |
| σ2 | 24.12 | | |
| τ00 Unique_Code | 26.79 | | |
| ICC | 0.53 | | |
| N Unique_Code | 44 | | |
| Observations | 528 | | |
| Marginal R2 / Conditional R2 | 0.330 / 0.683 | | |

**Table S27.** *Table reporting regression estimates, confidence intervals, and p-values of predictors for the model on VCS including Better Live as a man than as woman as* *a covariate.*

| **Predictors** | **Estimates** | **CI** | **p** |
| --- | --- | --- | --- |
| (Intercept) | 34.52 | 32.90 – 36.13 | <0.001 |
| Group1 | -4.22 | -5.84 – -2.61 | <0.001 |
| Condition1 | 2.37 | 1.77 – 2.97 | <0.001 |
| Condition2 | -0.59 | -1.20 – 0.01 | 0.055 |
| Better Live (man) | 0.13 | -0.84 – 1.11 | 0.789 |
| Group1 × Condition1 | 2.35 | 1.74 – 2.95 | <0.001 |
| Group1 × Condition2 | -0.96 | -1.57 – -0.36 | 0.002 |
| Group1 × Better Live (man) | 0.37 | -0.61 – 1.35 | 0.460 |
| Condition1 × Better Live (man) | -0.09 | -0.46 – 0.27 | 0.616 |
| Condition2 × Better Live (man) | -0.02 | -0.38 – 0.35 | 0.931 |
| (Group1 × Condition1) × Better Live (man) | -0.24 | -0.60 – 0.13 | 0.208 |
| (Group1 × Condition2) × Better Live (man) | 0.19 | -0.17 – 0.56 | 0.303 |
| Random Effects | | | |
| σ2 | 24.38 | | |
| τ00 Unique_Code | 26.90 | | |
| ICC | 0.52 | | |
| N Unique_Code | 44 | | |
| Observations | 528 | | |
| Marginal R2 / Conditional R2 | 0.325 / 0.679 | | |

**Table S28.** *Table reporting regression estimates, confidence intervals, and p-values of predictors for the model on VCS including Discrimination as* *a covariate.*

| **Predictors** | **Estimates** | **CI** | **p** |
| --- | --- | --- | --- |
| (Intercept) | 30.17 | 27.72 – 32.61 | <0.001 |
| Condition1 | 4.80 | 3.80 – 5.80 | <0.001 |
| Condition2 | -1.60 | -2.60 – -0.60 | 0.002 |
| Discrimination | -0.45 | -1.56 – 0.66 | 0.430 |
| Condition1 × Discrimination | -1.00 | -1.45 – -0.54 | <0.001 |
| Condition2 × Discrimination | 0.34 | -0.11 – 0.80 | 0.138 |
| Random Effects | | | |
| σ2 | 33.94 | | |
| τ00 Unique_Code | 31.14 | | |
| ICC | 0.48 | | |
| N Unique_Code | 22 | | |
| Observations | 264 | | |
| Marginal R2 / Conditional R2 | 0.192 / 0.579 | | |

**Table S29.** *Table reporting regression estimates, confidence intervals, and p-values of predictors for the model on VCS including Victimization as* *a covariate.*

| **Predictors** | **Estimates** | **CI** | **p** |
| --- | --- | --- | --- |
| (Intercept) | 30.17 | 27.78 – 32.55 | <0.001 |
| Condition1 | 4.80 | 3.76 – 5.83 | <0.001 |
| Condition2 | -1.60 | -2.63 – -0.56 | 0.003 |
| Victimization | 0.41 | -0.22 – 1.04 | 0.198 |
| Condition1 × Victimization | -0.14 | -0.42 – 0.13 | 0.304 |
| Condition2 × Victimization | 0.02 | -0.26 – 0.29 | 0.906 |
| Random Effects | | | |
| σ2 | 36.51 | | |
| τ00 Unique_Code | 29.29 | | |
| ICC | 0.45 | | |
| N Unique_Code | 22 | | |
| Observations | 264 | | |
| Marginal R2 / Conditional R2 | 0.182 / 0.546 | | |

**Table S30.** *Table reporting regression estimates, confidence intervals, and p-values of predictors for the model on VCS including Rejection as* *a covariate.*

| **Predictors** | **Estimates** | **CI** | **p** |
| --- | --- | --- | --- |
| (Intercept) | 30.17 | 27.69 – 32.64 | <0.001 |
| Condition1 | 4.80 | 3.76 – 5.84 | <0.001 |
| Condition2 | -1.60 | -2.64 – -0.56 | 0.003 |
| Rejection | 0.15 | -0.55 – 0.86 | 0.669 |
| Condition1 × Rejection | 0.11 | -0.19 – 0.40 | 0.481 |
| Condition2 × Rejection | -0.08 | -0.37 – 0.22 | 0.615 |
| Random Effects | | | |
| σ2 | 36.62 | | |
| τ00 Unique_Code | 31.66 | | |
| ICC | 0.46 | | |
| N Unique_Code | 22 | | |
| Observations | 264 | | |
| Marginal R2 / Conditional R2 | 0.153 / 0.546 | | |

**Table S31.** *Table reporting regression estimates, confidence intervals, and p-values of predictors for the model on VCS including Nonaffirmation as* *a covariate.*

| **Predictors** | **Estimates** | **CI** | **p** |
| --- | --- | --- | --- |
| (Intercept) | 30.17 | 28.10 – 32.23 | <0.001 |
| Condition1 | 4.80 | 3.77 – 5.83 | <0.001 |
| Condition2 | -1.60 | -2.63 – -0.56 | 0.003 |
| Nonaffirmation | -0.45 | -0.74 – -0.15 | 0.003 |
| Condition1 × Nonaffirmation | 0.10 | -0.05 – 0.25 | 0.180 |
| Condition2 × Nonaffirmation | -0.02 | -0.17 – 0.13 | 0.777 |
| Random Effects | | | |
| σ2 | 36.39 | | |
| τ00 Unique_Code | 21.14 | | |
| ICC | 0.37 | | |
| N Unique_Code | 22 | | |
| Observations | 264 | | |
| Marginal R2 / Conditional R2 | 0.278 / 0.543 | | |

**Table S32.** *Table reporting regression estimates, confidence intervals, and p-values of predictors for the model on VCS including Internalized Transphobia as* *a covariate.*

| **Predictors** | **Estimates** | **CI** | **p** |
| --- | --- | --- | --- |
| (Intercept) | 30.17 | 27.79 – 32.54 | <0.001 |
| Condition1 | 4.80 | 3.78 – 5.82 | <0.001 |
| Condition2 | -1.60 | -2.62 – -0.58 | 0.002 |
| Internalized Transphobia | -0.20 | -0.48 – 0.09 | 0.177 |
| Condition1 × Internalized Transphobia | 0.18 | 0.05 – 0.30 | 0.005 |
| Condition2 × Internalized Transphobia | -0.03 | -0.16 – 0.09 | 0.586 |
| Random Effects | | | |
| σ2 | 35.37 | | |
| τ00 Unique_Code | 29.14 | | |
| ICC | 0.45 | | |
| N Unique_Code | 22 | | |
| Observations | 264 | | |
| Marginal R2 / Conditional R2 | 0.198 / 0.560 | | |

**Table S33.** *Table reporting regression estimates, confidence intervals, and p-values of predictors for the model on VCS including Negative Expectations as* *a covariate.*

| **Predictors** | **Estimates** | **CI** | **p** |
| --- | --- | --- | --- |
| (Intercept) | 30.17 | 27.68 – 32.65 | <0.001 |
| Condition1 | 4.80 | 3.77 – 5.83 | <0.001 |
| Condition2 | -1.60 | -2.63 – -0.57 | 0.002 |
| Negative Expectations | 0.03 | -0.38 – 0.45 | 0.869 |
| Condition1 × Negative Expectations | 0.16 | -0.01 – 0.34 | 0.061 |
| Condition2 × Negative Expectations | -0.06 | -0.23 – 0.11 | 0.496 |
| Random Effects | | | |
| σ2 | 36.15 | | |
| τ00 Unique_Code | 31.97 | | |
| ICC | 0.47 | | |
| N Unique_Code | 22 | | |
| Observations | 264 | | |
| Marginal R2 / Conditional R2 | 0.155 / 0.552 | | |

**Table S34.** *Table reporting regression estimates, confidence intervals, and p-values of predictors for the model on VCS including Nondisclosure as* *a covariate.*

| Predictors | Estimates | CI | p |
| --- | --- | --- | --- |
| (Intercept) | 30.17 | 27.78 – 32.55 | <0.001 |
| Condition1 | 4.80 | 3.82 – 5.78 | <0.001 |
| Condition2 | -1.60 | -2.58 – -0.62 | 0.001 |
| Nondisclosure | -0.27 | -0.68 – 0.14 | 0.198 |
| Condition1 × Nondisclosure | 0.46 | 0.29 – 0.63 | <0.001 |
| Condition2 × Nondisclosure | -0.20 | -0.37 – -0.03 | 0.019 |
| Random Effects | | | |
| σ2 | 32.69 | | |
| τ00 Unique_Code | 29.61 | | |
| ICC | 0.48 | | |
| N Unique_Code | 22 | | |
| Observations | 264 | | |
| Marginal R2 / Conditional R2 | 0.225 / 0.593 | | |

**Table S35.** *Table reporting regression estimates, confidence intervals, and p-values of predictors for the model on VCS including Pride as* *a covariate.*

| **Predictors** | **Estimates** | **CI** | **p** |
| --- | --- | --- | --- |
| (Intercept) | 30.17 | 27.68 – 32.65 | <0.001 |
| Condition1 | 4.80 | 3.78 – 5.81 | <0.001 |
| Condition2 | -1.60 | -2.61 – -0.58 | 0.002 |
| Pride | -0.01 | -0.34 – 0.32 | 0.957 |
| Condition1 × Pride | -0.22 | -0.36 – -0.09 | 0.002 |
| Condition2 × Pride | 0.07 | -0.07 – 0.21 | 0.313 |
| Random Effects | | | |
| σ2 | 35.11 | | |
| τ00 Unique_Code | 32.10 | | |
| ICC | 0.48 | | |
| N Unique_Code | 22 | | |
| Observations | 264 | | |
| Marginal R2 / Conditional R2 | 0.167 / 0.565 | | |

**Table S36.** *Table reporting regression estimates, confidence intervals, and p-values of predictors for the model on VCS including Community Connectedness as* *a covariate.*

| **Predictors** | **Estimates** | **CI** | **p** |
| --- | --- | --- | --- |
| (Intercept) | 30.17 | 27.70 – 32.63 | <0.001 |
| Condition1 | 4.80 | 3.78 – 5.82 | <0.001 |
| Condition2 | -1.60 | -2.62 – -0.58 | 0.002 |
| Community Connectedness | -0.15 | -0.73 – 0.42 | 0.602 |
| Condition1 × Community Connectedness | -0.33 | -0.57 – -0.09 | 0.007 |
| Condition2 × Community Connectedness | 0.17 | -0.07 – 0.41 | 0.156 |
| Random Effects | | | |
| σ2 | 35.60 | | |
| τ00 Unique_Code | 31.59 | | |
| ICC | 0.47 | | |
| N Unique_Code | 22 | | |
| Observations | 264 | | |
| Marginal R2 / Conditional R2 | 0.166 / 0.558 | | |

**Table S37.** *Chi-squared tests, p-values, and FDR adjusted p-values for each term of each MAIA model.*

| **Model** | **Term** | **χ2** | **p** | **p adj** |
| --- | --- | --- | --- | --- |
| Attention Regulation | Group | 24.531 | < .001 | < .001 |
| Attention Regulation | Condition | 60.1 | < .001 | < .001 |
| Attention Regulation | Attention Regulation | 2.903 | .088 | .133 |
| Attention Regulation | Group:Condition | 52.831 | < .001 | < .001 |
| Attention Regulation | Group:Attention Regulation | 0.174 | .676 | .721 |
| Attention Regulation | Condition:Attention Regulation | 6.656 | .036 | .059 |
| Attention Regulation | Group:Condition:Attention Regulation | 4.832 | .089 | .133 |
| Body Listening | Group | 24.125 | < .001 | < .001 |
| Body Listening | Condition | 59.084 | < .001 | < .001 |
| Body Listening | Body Listening | 0.499 | .480 | .549 |
| Body Listening | Group:Condition | 59.455 | < .001 | < .001 |
| Body Listening | Group:Body Listening | 1.079 | .299 | .368 |
| Body Listening | Condition:Body Listening | 0.593 | .744 | .759 |
| Body Listening | Group:Condition:Body Listening | 1.404 | .496 | .556 |
| Emotional Awareness | Group | 27.819 | < .001 | < .001 |
| Emotional Awareness | Condition | 69.882 | < .001 | < .001 |
| Emotional Awareness | Emotional Awareness | 0.522 | .470 | .547 |
| Emotional Awareness | Group:Condition | 62.626 | < .001 | < .001 |
| Emotional Awareness | Group:Emotional Awareness | 0.096 | .756 | .759 |
| Emotional Awareness | Condition:Emotional Awareness | 18.817 | < .001 | < .001 |
| Emotional Awareness | Group:Condition:Emotional Awareness | 21.341 | < .001 | < .001 |
| Noticing | Group | 29.12 | < .001 | < .001 |
| Noticing | Condition | 68.702 | < .001 | < .001 |
| Noticing | Noticing | 0.266 | .606 | .658 |
| Noticing | Group:Condition | 64.008 | < .001 | < .001 |
| Noticing | Group:Noticing | 1.462 | .227 | .309 |
| Noticing | Condition:Noticing | 2.559 | .278 | .356 |
| Noticing | Group:Condition:Noticing | 0.552 | .759 | .759 |
| Not Distracting | Group | 33.616 | < .001 | < .001 |
| Not Distracting | Condition | 71.192 | < .001 | < .001 |
| Not Distracting | Not Distracting | 4.473 | .034 | .058 |
| Not Distracting | Group:Condition | 62.82 | < .001 | < .001 |
| Not Distracting | Group:Not Distracting | 0.619 | .431 | .511 |
| Not Distracting | Condition:Not Distracting | 1.338 | .512 | .565 |
| Not Distracting | Group:Condition:Not Distracting | 2.59 | .274 | .356 |
| Not Worrying | Group | 29.561 | < .001 | < .001 |
| Not Worrying | Condition | 61.64 | < .001 | < .001 |
| Not Worrying | Not Worrying | 2.523 | .112 | .163 |
| Not Worrying | Group:Condition | 57.443 | < .001 | < .001 |
| Not Worrying | Group:Not Worrying | 4.337 | .037 | .060 |
| Not Worrying | Condition:Not Worrying | 3.497 | .174 | .242 |
| Not Worrying | Group:Condition:Not Worrying | 8.415 | .015 | .026 |
| Self Regulation | Group | 16.552 | < .001 | < .001 |
| Self Regulation | Condition | 55.625 | < .001 | < .001 |
| Self Regulation | Self Regulation | 5.263 | .022 | .038 |
| Self Regulation | Group:Condition | 44.575 | < .001 | < .001 |
| Self Regulation | Group:Self Regulation | 1.226 | .268 | .356 |
| Self Regulation | Condition:Self Regulation | 0.747 | .688 | .722 |
| Self Regulation | Group:Condition:Self Regulation | 4.23 | .121 | .172 |
| Trusting | Group | 9.741 | .002 | .003 |
| Trusting | Condition | 11.183 | .004 | .007 |
| Trusting | Trusting | 1.078 | .299 | .368 |
| Trusting | Group:Condition | 5.069 | .079 | .124 |
| Trusting | Group:Trusting | 1.047 | .306 | .370 |
| Trusting | Condition:Trusting | 29.673 | < .001 | < .001 |
| Trusting | Group:Condition:Trusting | 19.91 | < .001 | < .001 |

**Table S38.** *Chi-squared tests, p-values, and FDR adjusted p-values for each term of each TAS-20 model.*

| **Model** | **Term** | **χ2** | **p** | **p adj** |
| --- | --- | --- | --- | --- |
| Difficulty Identifying Feeling | Group | 14.649 | < .001 | < .001 |
| Difficulty Identifying Feeling | Condition | 41.857 | < .001 | < .001 |
| Difficulty Identifying Feeling | Difficulty Identifying Feeling | 1.587 | .208 | .262 |
| Difficulty Identifying Feeling | Group:Condition | 24.047 | < .001 | < .001 |
| Difficulty Identifying Feeling | Group:Difficulty Identifying Feeling | 2.701 | .100 | .142 |
| Difficulty Identifying Feeling | Condition:Difficulty Identifying Feeling | 9.322 | .009 | .017 |
| Difficulty Identifying Feeling | Group:Condition:Difficulty Identifying Feeling | 0.579 | .749 | .781 |
| Difficulty Describing Feelings | Group | 26.245 | < .001 | < .001 |
| Difficulty Describing Feelings | Condition | 64.512 | < .001 | < .001 |
| Difficulty Describing Feelings | Difficulty Describing Feelings | 0.679 | .410 | .487 |
| Difficulty Describing Feelings | Group:Condition | 42.546 | < .001 | < .001 |
| Difficulty Describing Feelings | Group:Difficulty Describing Feelings | 6.344 | .012 | .020 |
| Difficulty Describing Feelings | Condition:Difficulty Describing Feelings | 5.294 | .071 | .106 |
| Difficulty Describing Feelings | Group:Condition:Difficulty Describing Feelings | 0.663 | .718 | .781 |
| Externally-Oriented Thinking | Group | 28.611 | < .001 | < .001 |
| Externally-Oriented Thinking | Condition | 72.267 | < .001 | < .001 |
| Externally-Oriented Thinking | Externally-Oriented Thinking | 0.001 | .970 | .970 |
| Externally-Oriented Thinking | Group:Condition | 63.702 | < .001 | < .001 |
| Externally-Oriented Thinking | Group:Externally-Oriented Thinking | 2.278 | .131 | .175 |
| Externally-Oriented Thinking | Condition:Externally-Oriented Thinking | 1.705 | .426 | .487 |
| Externally-Oriented Thinking | Group:Condition:Externally-Oriented Thinking | 7.731 | .021 | .034 |

**Table S39.** *Chi-squared tests, p-values, and FDR adjusted p-values for each term of each ERQ model.*

| **Model** | **Term** | **χ2** | **p** | **p adj** |
| --- | --- | --- | --- | --- |
| Cognitive Reappraisal | Group | 26.028 | < .001 | < .001 |
| Cognitive Reappraisal | Condition | 71.543 | < .001 | < .001 |
| Cognitive Reappraisal | Cognitive Reappraisal | 3.385 | .066 | .117 |
| Cognitive Reappraisal | Group:Condition | 63.391 | < .001 | < .001 |
| Cognitive Reappraisal | Group:Cognitive Reappraisal | 0.118 | .732 | .780 |
| Cognitive Reappraisal | Condition:Cognitive Reappraisal | 1.885 | .390 | .520 |
| Cognitive Reappraisal | Group:Condition:Cognitive Reappraisal | 3.447 | .178 | .286 |
| Expressive Suppression | Group | 28.534 | < .001 | < .001 |
| Expressive Suppression | Condition | 64.766 | < .001 | < .001 |
| Expressive Suppression | Expressive Suppression | 0.835 | .361 | .520 |
| Expressive Suppression | Group:Condition | 55.106 | < .001 | < .001 |
| Expressive Suppression | Group:Expressive Suppression | 0.317 | .573 | .655 |
| Expressive Suppression | Condition:Expressive Suppression | 1.543 | .462 | .569 |
| Expressive Suppression | Group:ConditionExpressive Suppression | 0.231 | .891 | .891 |

**Table S40.** *Chi-squared tests, p-values, and FDR adjusted p-values for each term of each MULTI-GIQ model.*

| **Model** | **Term** | **χ2** | **p** | **p adj** |
| --- | --- | --- | --- | --- |
| Better Live (man) | Group | 26.513 | < .001 | < .001 |
| Better Live (man) | Condition | 64.335 | < .001 | < .001 |
| Better Live (man) | Better Live (man) | 0.072 | .789 | .871 |
| Better Live (man) | Group:Condition | 58.942 | < .001 | < .001 |
| Better Live (man) | Group:Better Live (man) | 0.547 | .460 | .649 |
| Better Live (man) | Condition:Better Live (man) | 0.404 | .817 | .877 |
| Better Live (man) | Group:Condition:Better Live (man) | 1.802 | .406 | .627 |
| Better Live (woman) | Group | 22.987 | < .001 | < .001 |
| Better Live (woman) | Condition | 57.969 | < .001 | < .001 |
| Better Live (woman) | Better Live (woman) | 0.818 | .366 | .619 |
| Better Live (woman) | Group:Condition | 49.332 | < .001 | < .001 |
| Better Live (woman) | Group:Better Live (woman) | 0.069 | .793 | .871 |
| Better Live (woman) | Condition:Better Live (woman) | 3.961 | .138 | .270 |
| Better Live (woman) | Group:Condition:Better Live (woman) | 2.048 | .359 | .619 |
| Dislike Sexed Body (male) | Group | 0.751 | .386 | .623 |
| Dislike Sexed Body (male) | Condition | 6.167 | .046 | .096 |
| Dislike Sexed Body (male) | Dislike Sexed Body (male) | 0.261 | .610 | .745 |
| Dislike Sexed Body (male) | Group:Condition | 0.981 | .612 | .745 |
| Dislike Sexed Body (male) | Group: Dislike Sexed Body (male) | 0.213 | .645 | .756 |
| Dislike Sexed Body (male) | Condition: Dislike Sexed Body (male) | 1.618 | .445 | .649 |
| Dislike Sexed Body (male) | Group:Condition: Dislike Sexed Body (male) | 0.232 | .890 | .927 |
| Dislike Sexed Body (female) | Group | 8.528 | .003 | .008 |
| Dislike Sexed Body (female) | Condition | 14.92 | .001 | .001 |
| Dislike Sexed Body (female) | Dislike Sexed Body (female) | 1.553 | .213 | .382 |
| Dislike Sexed Body (female) | Group:Condition | 19.541 | < .001 | < .001 |
| Dislike Sexed Body (female) | Group: Dislike Sexed Body (female) | 1.894 | .169 | .316 |
| Dislike Sexed Body (female) | Condition: Dislike Sexed Body (female) | 0.442 | .802 | .871 |
| Dislike Sexed Body (female) | Group:Condition: Dislike Sexed Body (female) | 0.921 | .631 | .750 |
| Wish Other Sexed Body | Group | 7.317 | .007 | .015 |
| Wish Other Sexed Body | Condition | 8.921 | .012 | .025 |
| Wish Other Sexed Body | Wish Body (Wish Other Sexed Body) | 0.285 | .594 | .745 |
| Wish Other Sexed Body | Group:Condition | 1.617 | .445 | .649 |
| Wish Other Sexed Body | Group:Wish Body (Wish Other Sexed Body) | 0.703 | .402 | .627 |
| Wish Other Sexed Body | Condition:Wish Body (Wish Other Sexed Body) | 5.735 | .057 | .116 |
| Wish Other Sexed Body | Group:Condition:Wish Body (Wish Other Sexed Body) | 1.172 | .557 | .742 |
| Pressure (man) | Group | 11.034 | .001 | .002 |
| Pressure (man) | Condition | 26.178 | < .001 | < .001 |
| Pressure (man) | Pressure (man) | 0 | .995 | .995 |
| Pressure (man) | Group:Condition | 14.467 | .001 | .002 |
| Pressure (man) | Group:Pressure (man) | 2.838 | .092 | .184 |
| Pressure (man) | Condition:Pressure (man) | 1.025 | .599 | .745 |
| Pressure (man) | Group:Condition:Pressure (man) | 3.486 | .175 | .321 |
| Pressure (woman) | Group | 12.7 | < .001 | .001 |
| Pressure (woman) | Condition | 54.379 | < .001 | < .001 |
| Pressure (woman) | Pressure (woman) | 1.995 | .158 | .302 |
| Pressure (woman) | Group:Condition | 39.952 | < .001 | < .001 |
| Pressure (woman) | Group:Pressure (woman) | 0.955 | .328 | .578 |
| Pressure (woman) | Condition:Pressure (woman) | 0.446 | .800 | .871 |
| Pressure (woman) | Group:Condition:Pressure (woman) | 1.591 | .451 | .649 |
| Satisfaction Affirmed Gender (men) | Group | 24.582 | < .001 | < .001 |
| Satisfaction Affirmed Gender (men) | Condition | 30.322 | < .001 | < .001 |
| Satisfaction Affirmed Gender (men) | Satisfaction Gender (man) | 0.641 | .423 | .642 |
| Satisfaction Affirmed Gender (men) | Group:Condition | 23.312 | < .001 | < .001 |
| Satisfaction Affirmed Gender (men) | Group:Satisfaction Gender (man) | 0.279 | .597 | .745 |
| Satisfaction Affirmed Gender (men) | Condition:Satisfaction Gender (man) | 0.26 | .878 | .927 |
| Satisfaction Affirmed Gender (men) | Group:Condition:Satisfaction Gender (man) | 0.168 | .920 | .941 |
| Satisfaction Affirmed Gender (women) | Group | 13.787 | < .001 | .001 |
| Satisfaction Affirmed Gender (women) | Condition | 55.977 | < .001 | < .001 |
| Satisfaction Affirmed Gender (women) | Satisfaction Gender (woman) | 0.767 | .381 | .623 |
| Satisfaction Affirmed Gender (women) | Group:Condition | 45.767 | < .001 | < .001 |
| Satisfaction Affirmed Gender (women) | Group:Satisfaction Gender (woman) | 0.017 | .896 | .927 |
| Satisfaction Affirmed Gender (women) | Condition:Satisfaction Gender (woman) | 1.532 | .465 | .649 |
| Satisfaction Affirmed Gender (women) | Group:Condition:Satisfaction Gender (woman) | 0.117 | .943 | .954 |
| Wish Other Gender (men) | Group | 21.825 | < .001 | < .001 |
| Wish Other Gender (men) | Condition | 46.693 | < .001 | < .001 |
| Wish Other Gender (men) | Wish Other Gender (man) | 0.445 | .505 | .683 |
| Wish Other Gender (men) | Group:Condition | 25.99 | < .001 | < .001 |
| Wish Other Gender (men) | Group:Wish Other Gender (men) | 6.018 | .014 | .030 |
| Wish Other Gender (men) | Condition:Wish Other Gender (men) | 1.093 | .579 | .745 |
| Wish Other Gender (men) | Group:Condition:Wish Other Gender (men) | 0.659 | .719 | .833 |
| Wish Other Gender (women) | Group | 8.587 | .003 | .008 |
| Wish Other Gender (women) | Condition | 25.718 | < .001 | < .001 |
| Wish Other Gender (women) | Wish Other Gender (women) | 0.469 | .493 | .679 |
| Wish Other Gender (women) | Group:Condition | 31.624 | < .001 | < .001 |
| Wish Other Gender (women) | Group:Wish Other Gender (women) | 0.741 | .389 | .623 |
| Wish Other Gender (women) | Condition:Wish Other Gender (women) | 0.455 | .796 | .871 |
| Wish Other Gender (women) | Group:Condition:Wish Other Gender (women) | 0.964 | .618 | .745 |

**Table S41.** *Chi-squared tests, p-values, and FDR adjusted p-values for each term of each GMSR model.*

| **Model** | **Term** | **χ2** | **p** | **p adj** |
| --- | --- | --- | --- | --- |
| Community Connectedness | Condition | 88.564 | < .001 | < .001 |
| Community Connectedness | Community Connectedness | 0.273 | .601 | .676 |
| Community Connectedness | Condition:Community Connectedness | 7.327 | .026 | .038 |
| Discrimination | Condition | 92.915 | < .001 | < .001 |
| Discrimination | Discrimination | 0.625 | .429 | .515 |
| Discrimination | Condition:Discrimination | 19.379 | < .001 | < .001 |
| Internalized Transphobia | Condition | 89.161 | < .001 | < .001 |
| Internalized Transphobia | Internalized Transphobia | 1.832 | .176 | .244 |
| Internalized Transphobia | Condition:Internalized Transphobia | 8.98 | .011 | .018 |
| Negative Expectations | Condition | 87.23 | < .001 | < .001 |
| Negative Expectations | Negative Expectations | 0.027 | .869 | .894 |
| Negative Expectations | Condition:Negative Expectations | 3.632 | .163 | .234 |
| Nonaffirmation | Condition | 86.646 | < .001 | < .001 |
| Nonaffirmation | Nonaffirmation | 8.987 | .003 | .005 |
| Nonaffirmation | Condition:Nonaffirmation | 2.012 | .366 | .454 |
| Nondisclosure | Condition | 96.456 | < .001 | < .001 |
| Nondisclosure | Nondisclosure | 1.665 | .197 | .253 |
| Nondisclosure | Condition:Nondisclosure | 29.187 | < .001 | < .001 |
| Pride | Condition | 89.8 | < .001 | < .001 |
| Pride | Pride | 0.003 | .957 | .957 |
| Pride | Condition:Pride | 10.75 | .005 | .008 |
| Rejection | Condition | 86.11 | < .001 | < .001 |
| Rejection | Rejection | 0.183 | .669 | .729 |
| Rejection | Condition:Rejection | 0.528 | .768 | .813 |
| Victimization | Condition | 86.378 | < .001 | < .001 |
| Victimization | Victimization | 1.668 | .196 | .253 |
| Victimization | Condition:Victimization | 1.27 | .530 | .615 |
